# Parent-Guided Developmental Intervention for Infants With Very Low Birth Weight: A Randomized Clinical Trial

**DOI:** 10.1001/jamanetworkopen.2024.21896

**Published:** 2024-07-17

**Authors:** Rita C. Silveira, Nadia C. Valentini, T. Michael O’Shea, Eliane W. Mendes, Graciela Froes, Lenir Cauduro, Carolina Panceri, Rubia N. Fuentefria, Renato S. Procianoy

**Affiliations:** 1 Neonatal Section, Hospital de Clínicas de Porto Alegre, Porto Alegre, Brazil; 2 Programa de Pós-Graduação em Saúde da Criança e Adolescente, Federal University of Rio Grande do Sul, Porto Alegre, Brazil; 3 Department of Pediatrics, The University of North Carolina at Chapel Hill

## Abstract

**Importance:**

Early interventions improve neurodevelopmental outcomes after preterm birth, but few studies of early intervention have focused on preterm infants whose families reside in low- or middle-income countries (LMICs).

**Objective:**

To evaluate whether parent-guided early intervention improves the neurodevelopmental outcomes of preterm infants in an LMIC.

**Design, Setting, and Participants:**

This randomized clinical trial was performed at a high-risk obstetric referral hospital in Brazil, with outcome evaluations by examiners masked to randomization group. Eligibility criteria were (1) birth at the study hospital, (2) residence within 40 km of the birth hospital, and (3) gestational age of less than 32 weeks or birth weight of less than 1500 g. Of 138 enrolled infants, 19 died after randomization and 19 withdrew from the study; all other enrollees (50 per randomization group) were evaluated for the primary outcome. Data were collected from January 1, 2016, to May 31, 2022, and analyzed from June 10 to July 31, 2022.

**Interventions:**

On postnatal day 7, infants were randomized to usual care, consisting of support for lactation, kangaroo care, and routine developmental therapies, or to a parent-guided enhanced developmental intervention, consisting of usual care plus infant massage and enhanced visual stimulation, auditory stimulation, social interactions, and support for motor development, instructed by developmental therapists.

**Main Outcomes and Measures:**

The primary outcome was the Bayley Scales of Infant and Toddler Development–Third Edition score at 18 months of age adjusted for prematurity.

**Results:**

Among the 100 infants included in the analysis, mean (SD) gestational age was 28.4 (2.2) weeks, and 57 (57%) were male. The mean (SD) gestational age for the intervention group was 28.3 (2.3) weeks; for the usual care group, 28.5 (2.2) weeks. Female infants accounted for 21 infants (42%) of the intervention group and 22 (44%) of the usual care group; male infants, 29 (58%) and 28 (56%), respectively. The enhanced developmental intervention group had higher cognitive scores at 18 months of corrected age (mean [SD], 101.8 [11.9] vs 97.3 [13.5]; mean difference, 4.5 [95% CI, 0.1-8.9]).

**Conclusions and Relevance:**

In this randomized clinical trial of a parent-guided developmental intervention for early cognitive function of very preterm or very low birth weight infants implemented in an LMIC, the intervention improved very preterm infants’ neurodevelopmental outcomes at 18 months of adjusted age. Parent-guided early intervention can improve neurodevelopmental outcome of very preterm infants born in LMICs.

**Trial Registration:**

ClinicalTrials.gov Identifier: NCT02835612

## Key Points

**Question:**

In low- to middle-income countries (LMICs), does a parent-guided early intervention for very preterm infants or those with very low birth weight improve cognitive outcomes at 18 months of age compared with usual care?

**Findings:**

In this randomized clinical trial among 100 infants of families residing in an LMIC, the parent-guided developmental intervention initiated in the neonatal intensive care unit and continued in the home improved early cognitive function of very preterm or very low birth weight infants.

**Meaning:**

Parent-guided early intervention can improve neurodevelopmental outcome of very preterm or very low birth weight infants born in LMICs.

## Teaser

This randomized clinical trial describes the effects of a developmentally supportive intervention designed to improve neurodevelopmental outcomes among infants born very low birth weight or very preterm in a low- to middle-income country, compared with usual care.

## Introduction

Preterm birth and socioeconomic disadvantage are strong predictive factors associated with neurodevelopmental impairment, and the risk of such impairments can be improved by interventions to support infant neurodevelopment when implemented during the neonatal hospitalization^1^ or post hospital discharge.^2^ Early interventions that involve education and/or support for parents can improve parents’ psychosocial well-being and thereby improve outcomes of the infant.^3^ Few studies of early intervention for preterm infants have focused on parents who have low socioeconomic resources and reside in low- to middle-income countries (LMICs).^4,5,6^

Herein we describe the effects of a developmentally supportive intervention designed to improve neurodevelopmental outcomes among infants born with very low birth weight or very preterm whose families resided in Brazil, an LMIC. This intervention included periodic encounters between developmental specialists and parents with the goals of providing emotional support for the parents, modeling developmentally supportive activities for infants, sensitizing parents to their infants’ cues, and promoting appropriate responses to their infants’ needs by educational instructions and modeling of behaviors. We posited that increasing parents’ psychological well-being, the quality of parent-infant interactions, and developmentally supportive activities would enhance infants’ regulatory abilities, brain plasticity, and cognitive and social development. In addition, therapists instructed parents in infant massage, passive stretching of extremities, and postural positioning, each of which could enhance the parents’ sense of parenting and infants’ motor development. We hypothesized that compared with usual care (control group), enhanced developmental supports, when initiated in the neonatal intensive care unit (NICU) and continued in the home during infancy, would improve infant neurodevelopmental as well as parent-infant interactions.

## Methods

When reporting the results of this randomized clinical trial, which was retrospectively registered at ClinicalTrials.gov after we were unable to register on the Brazilian Clinical Trials Registry, we followed the Consolidated Standards of Reporting Trials (CONSORT) reporting guideline. The institutional review board of Hospital de Clínicas de Porto Alegre, Porto Alegre, Brazil, approved the study, and participating parents or caregivers provided written informed consent.

### Study Design

We have previously published details of this randomized comparison of enhanced developmental intervention vs usual care, as well as strategies that were used to promote participant enrollment and neurodevelopmental follow-up.^7^ Participants and clinicians caring for participants in the NICU at the study institution were aware of their randomization assignments; individuals who evaluated the primary outcome, the Bayley Scales of Infant and Toddler Development–Third Edition (BSID-III) score, were masked to randomization assignment. When designing the study, we followed the Standard Protocol Items: Recommendations for Interventional Trials 2013 statement.^8^ The trial protocol is available in Supplement 1.

### Eligibility Criteria

Eligibility criteria included preterm infants with gestational age of less than 32 weeks or birth weight of less than 1500 g born at the study institution and residence within 40 km of the birth hospital. Exclusion criteria comprised major congenital malformations or inborn errors of metabolism; congenital infections, including syphilis, toxoplasmosis, varicella-zoster virus, parvovirus B19, rubella, cytomegalovirus, herpes simplex virus, and HIV; or autoimmune conditions. A sample size of 100 was selected toward the goal of obtaining the primary outcome, the BSID-III score, for at least 84 study participants. This sample size was estimated to provide 80% power to detect a difference of 3 points (equal to 0.2 SDs in the normative sample) in BSID-III scores.^9,10,11^ To allow for sample attrition, we randomized 20% more study participants than the targeted sample size, for a total of 100 mother-infant dyads.

The Figure presents the CONSORT diagram and flowchart showing the flow of participants through each stage of the randomized clinical trial. From January 1, 2016, to February 29, 2020, among eligible inborn preterm infants, 138 were randomized, of whom 100 completed the study protocol. Among the 38 randomized participants who did not complete the protocol, 15 died in the NICU, 4 died after discharge, and 19 did not complete all study procedures due to circumstances related to the COVID-19 pandemic (5 due to transfer to other city and 4 due to change of caregiver). Follow-up evaluations through 18 months of adjusted age were performed between November 1, 2017, and May 31, 2022; and initial data analyses were completed from June 10 to July 31, 2022.

**Figure.**
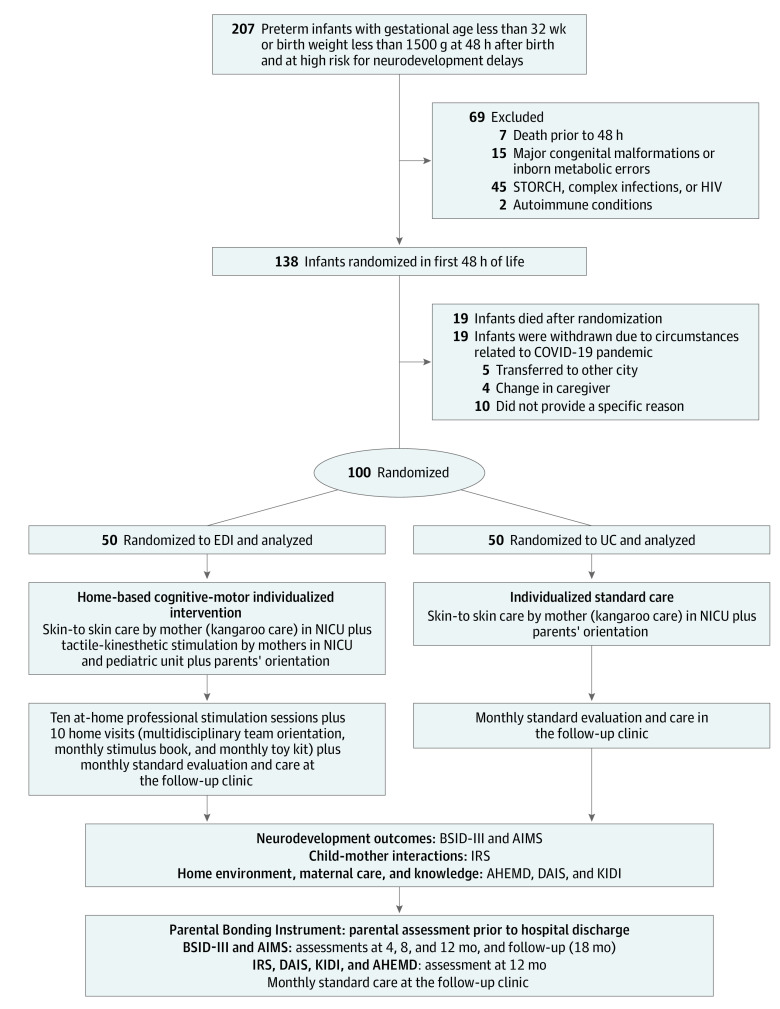
Study Flowchart AHEMD indicates Affordance in the Home Environment for Motor Development; AIMS, Alberta Infant Motor Scale; BSID-III, Bayley Scales of Infant and Toddler Development–Third Edition; DAIS, Daily Assessment Infant Scale; EDI, educational development intervention; IRS, Interaction Rating Scale; KIDI, Knowledge of Infant Development Inventory; NICU, neonatal intensive care unit; STORCH, congenital infections, including syphilis, toxoplasmosis, other agents (varicella-zoster virus and parvovirus B19), rubella, cytomegalovirus, and herpes simplex virus; and UC, usual care.

### Randomization

When infants reached 48 postnatal hours, their parents were recruited for enrollment. If the parents provided informed consent, their infant was randomized to either the enhanced developmental intervention or usual care using block randomization with computer-generated randomization assignments^8^ using the software program Block Randomization With Random Block Sizes from Vanderbilt University Medical Center.^12^

### Usual Care

Infants in the usual care group, who served as controls, received kangaroo care according to the routine of the NICU.^7^ After discharge from the NICU, infants in the usual care group were scheduled for routine visits to the follow-up clinic, where their developmental competencies were evaluated. Visits were scheduled monthly until 6 months of age adjusted for prematurity (referred to hereafter as adjusted age), bimonthly from 7 to 12 months of adjusted age, and every 3 months thereafter until 24 months of age. If a developmental delay was detected, the infant was referred for early intervention services provided by the Brazilian Unified Health System. Participants in both the usual care and enhanced developmental intervention groups were cared for in an open-bay NICU.

### Enhanced Developmental Intervention

Prior to initiation of this randomized clinical trial, the research team responsible for implementing the enhanced developmental intervention completed 20 training sessions designed to increase team members’ knowledge of effective techniques for educating parents about infant development and strategies for enhancing infant development, such as tactile-kinesthetic stimulation.^7,13^ In addition to receiving the aforementioned interventions provided to the usual care group, infants randomized to the enhanced developmental intervention group received educational activities for the parents consisting of instructions in how to provide their infant with tactile-kinesthetic stimulation and kangaroo care, provided to parents on alternate days and beginning on postnatal day 7.^7^

After hospital discharge, a multidisciplinary research team provided a home-based intervention for infants in the enhanced developmental intervention group to reinforce education that was provided previously in the NICU and to provide support and encouragement for caregivers and parents. At each of 10 home visits, the research team assessed the mothers’ comprehension of the guidelines and tasks, used illustrated folders to guide the mothers as to how to implement the tasks, and recorded how the intervention was being delivered by the families. These observations were used to counsel families and enhance adherence to the program.^7^ The multidisciplinary research team also explained to parents how the interventions are thought to influence clinical and developmental outcomes, encouraged and supported parents, and answered parents’ questions about their infants’ health and development. The family educational approach was designed for adult education oriented toward transformational and experiential learning.

### Measurements

#### Baseline Characteristics of Mothers and Families

Prior to the hospital discharge, parents of study participants completed a survey of socioeconomic factors, such as years of education, total household income, family composition, receipt of financial support from the Brazilian government, and the Parental Bonding Instrument^8^ to assess their own childhood experiences with their own parents. The Parental Bonding Instrument consists of 25 items and yields subscale scores for care and overprotection. For the care domain, scores can range from 0 to 36, with higher scores indicating more loving parental behavior, and lower scores indicating an indifferent or rejecting parental attitude. For the overprotection domain, scores can range from 0 to 39, with higher scores indicating overprotective or overly interfering parental behavior, and lower scores indicating a parental attitude that values spontaneity and child autonomy.^7^ Race and ethnicity data were not collected because all study participants were Brazilian.

#### Primary Outcome

Data about neonatal complications and therapies were collected by reviewing medical records. Infant neurodevelopmental outcomes were assessed using the BSID-III and the Alberta Infant Motor Scale (AIMS), administered at 4, 8, 12, and 18 months of age, adjusted for degree of prematurity. The primary outcome was BSID-III score at 18 months of adjusted age, administered by 1 of 2 examiners (N.C.V. and C.P.) who were masked to randomization assignment and had more than 5 years of experience administering the BSID-III. The BSID-III and AIMS were completed in the infant’s home with a parent or legal guardian present.

#### Secondary Outcomes

Secondary outcomes included child neurodevelopmental outcomes and child interactions, as well as measures of the home environment and maternal practices that we hypothesized would be improved in the enhanced developmental intervention compared with the usual care groups. Secondary infant neurodevelopmental outcomes were the BSID-III language and motor scales and AIMS. High interrater reliability levels were obtained (BSID-III, 96%; AIMS, 98%). From BSID-III scores, we derived binary outcomes of delayed cognitive, language, or motor development, defined as a score below 80. For the AIMS, a delay was defined as a score at less than 5.

Secondary outcomes related to the home environment and maternal practices were assessed at 12 months of adjusted age and included the Interaction Rating Scale, the Affordance in the Home Environment for Motor Development–Infant Scale, the Daily Activities of Infant Scale, and the Knowledge of Infant Development Inventory adapted for Brazilian children. To evaluate the quality of parent-infant interaction, we used the Interaction Rating Scale, which assesses mother-infant interactions based during a 10-minute observation of the child and mother interacting in a familiar environment, with a table, seats, and toys available for the child, after instructing the mother to interact with the child as she would typically do. The Interaction Rating Scale includes 70 items about behaviors and yields 10 subscales; 5 subscales (25 items) focus on children’s social skills (ie, autonomy, responsiveness, empathy, motor regulation, and emotional regulation); the other 5 (45 dichotomous items) focus on the caregiver’s parenting skills (ie, respect for autonomy development, respect for responsiveness development, respect for empathy development, respect for cognitive development, and respect for social-emotional development). An overall score of synchronous interactions between mother and the child is also measured.^14^ The Affordance in the Home Environment for Motor Development–Infant Scale evaluates development opportunities available at home and considers physical spaces (outside and inside the home), daily activities, and play materials. Higher scores indicate more enriched opportunities, with scores 0 to 18 indicating less than adequate; 19 to 23, moderately adequate; 24 to 27, adequate; and 28 to 49, excellent.^15^ The Daily Activities of Infant Scale assesses parental practices based on parents’ responses to questions about infant positions usually involved in baby care (eg, bathing and dressing) and sleeping (ie, supine, side, or prone position), with higher scores indicative of more favorable activities. The Knowledge of Infant Development Inventory assesses parental knowledge regarding infant development based on 20 questions about the age at which infants develop specific skills.^16^

### Statistical Analysis

Descriptive statistics included means and SDs for continuous variables and frequencies and percentages for categorical variables. Group comparisons of continuous variables were analyzed using 1-way analysis of variance. For comparisons of categorical variables, we used χ^2^ tests, and to describe associations we presented mean differences or relative risks and respective 95% CIs according to the variable analyzed. Two-sided *P* ≤ .05 indicated statistical significance. All analyses were performed using SPSS Statistic Data Editor, version 29 (IBM Corp).

## Results

### Participants

Attributes of the 100 study participants are summarized in Table 1. The mean (SD) gestational age for the intervention group (n = 50) was 28.3 (2.3) weeks, and for the usual care group (n = 50), 28.5 (2.2) weeks. The mean (SD) birth weight was 1115 (286) g for the intervention group and 1077 (318) g for the usual care group. Female infants accounted for 21 participants (42%) in the intervention group and 22 (44%) in the usual care group; male infants, 29 (58%) and 28 (56%), respectively.

**Table 1.** Family, Maternal, and Neonatal Variables for Study Participants

Variable	EDI group (n = 50)	UC group (n = 50)
**Family**		
Mother’s age at delivery, mean (SD), y	27.6 (7.0)	28.9 (6.7)
Father’s age at delivery, mean (SD), y	30.7 (7.0)	30.6 (7.2)
Family monthly income, mean (SD), US $	436 (280)	419 (313)
BFP governmental assistance, No. (%)^a^	6 (12)	7 (14)
No. of children at home, median (IQR)	1 (0-2)	1 (0-2)
No. of adolescents at home, median (IQR)	0 (0-1)	0 (0-1)
Parents’ report of their own parents’ parenting style, mean (SD)^b^		
Mother care	19.6 (5.7)	18.0 (4.9)
Mother overprotection	18.9 (5.9)	16.7 (6.5)
Father care	18.4 (5.3)	17.0 (4.1)
Father overprotection	18.3 (5.2)	16.0 (6.4)
Maternal educational level, No. (%)		
Incomplete middle school	6 (12)	7 (14)
Complete middle school	6 (12)	13 (26)
Incomplete high school	5 (10)	4 (8)
High school degree	21 (42)	16 (32)
Incomplete undergraduate	7 (14)	6 (12)
Undergraduate degree	5 (10)	2 (4)
Paternal educational level, No. (%)		
Incomplete middle school	9 (18)	8 (16)
Complete middle school	7 (14)	14 (28)
Incomplete high school	5 (10)	5 (10)
High school degree	20 (40)	22 (44)
Incomplete undergraduate	2 (4)	0
Undergraduate degree	6 (12)	3 (6)
Graduate degree	1 (2)	0
Parents live together, No. (%)		
Yes	39 (78)	43 (86)
No	11 (22)	7 (14)
Primary caregiver, No. (%)^c^		
Mother	45 (90)	48 (94)
Father	2 (4)	0
Grandparents	2 (4)	2 (4)
Other	1 (2)	1 (2)
**Maternal **		
No. of previous gestations, mean (SD)	2.2 (1.9)	2.4 (1.7)
No. of prenatal care visits, mean (SD)	5.1 (3.0)	4.5 (2.4)
Preeclampsia, No. (%)	14 (28)	18 (36)
Twin gestation, No. (%)	13 (26)	18 (36)
Breastfeeding at discharge, No. (%)	39 (78)	34 (68)
Cesarean delivery, No. (%)	34 (68)	37 (74)
**Neonatal**		
Sex, No. (%)		
Female	21 (42)	22 (44)
Male	29 (58)	28 (56)
Gestational age, mean (SD), wk	28.3 (2.3)	28.5 (2.2)
Birth weight, mean (SD), g	1115 (286)	1077 (318)
Length at birth, mean (SD), cm	37.2 (3.4)	35.9 (4.2)
Small for gestational age, No. (%)	12 (24)	11 (22)
Head circumference at birth, mean (SD) cm	26.1 (2.5)	25.6 (3.5)
Apgar score, median (IQR), min		
1	6 (4-8)	6 (4-8)
5	8 (7-9)	8 (7-9)
Hemoglobin levels at discharge, mean (SD), mg/dL	8.6 (0.5)	8.5 (0.8)
No. of additional hospital admissions after NICU discharge during the study, median (IQR)	2 (0-3)	2 (1-4)

Abbreviations: BFP, Bolsa Familia Program; EDI, educational development intervention; NICU, neonatal intensive care unit; UC, usual care.

SI conversion factor: To convert hemoglobin to g/L, multiply by 0.1.

^a^ Indicates a government conditional cash transfer program for poor families (maximum income US $70 per person/month) when they meet conditions related to health and education. Governmental monetary support ranges from US $18 to $175 per month, depending on the income and family composition.

^b^ For the Care domain, scores range from 0 to 36, with higher scores indicating a more loving parental behavior, and lower scores indicating an indifferent or rejecting parental attitude. For the Overprotection domain, scores range from 0 to 39, with higher scores indicating overprotective or overly interfering parental behavior, and lower scores indicating a parental attitude that values spontaneity and child autonomy.^7^

^c^ In the UC group, 1 patient changed from the mother to the grandmother during the study.

The enhanced developmental intervention and usual care groups were similar with respect to maternal, family, and neonatal characteristics (Table 1). Among the 100 study participants who completed the study protocol, the mean (SD) birth weight was 1096 (300) g, and the mean (SD) gestational age was 28.4 (2.2) weeks. Among mothers, 48 (48%) had no formal education beyond high school. eTable 1 in Supplement 2 summarizes neonatal complications, which were similar in the enhanced developmental intervention and usual care groups. Overall, 14 infants (14%) had bronchopulmonary dysplasia, 32 (32%) had late-onset sepsis, and 35 (35%) had periventricular hemorrhage.

### Primary Outcome

Table 2 compares BSID-III cognitive, language, and motor scores and AIMS scores and frequencies of developmental delay for the enhanced developmental intervention and usual care groups. The primary outcome, the BSID-III score at 18 months of corrected age was higher in the enhanced developmental intervention group (mean [SD], 101.8 [11.9] vs 97.3 [13.5]; mean difference, 4.5 [95% CI, 0.1-8.9]).

**Table 2.** Comparison of BSID-III Cognitive, Language, and Motor Scores and AIMS Scores at 18 Months of Age by Randomization Group^a^

Measure	Study group, mean (SD)		Mean difference (95% CI)	Relative risk (95% CI)
	EDI (n = 50)	UC (n = 50)		
BSID-III cognitive score	101.8 (11.9)	97.3 (13.5)	4.5 (0.1-8.9)	NA
BSID-III language score	99.3 (12.0)	91.6 (14.4)	7.7 (2.1-13.3)	NA
BSID-III motor score	102.5 (13.3)	92.7 (17.9)	9.8 (3.2-16.5)	NA
AIMS score	53.7 (17.0)	30.3 (21.1)	23.4 (16.8-30.0)	NA
BSID-III cognitive delay, No. (%)	4 (8)	6 (12)	NA	0.7 (0.2-1.8)
BSID-III language delay, No. (%)	14 (28)	17 (34)	NA	0.4 (0.2-0.7)
BSID-IIII motor delay, No. (%)	9 (18)	16 (32)	NA	0.4 (0.2-0.9)
AIMS motor delay, No. (%)	1 (2)	15 (30)	NA	0.1 (0-0.8)

Abbreviations: AIMS, Alberta Infant Motor Scale; BSID-III, Bayley Scales of Infant and Toddler Development–Third Edition; EDI, educational development intervention; NA, not applicable; UC, usual care.

^a^ BSID-III scores range from 46 to 154, with lower scores indicating more delay and higher scores indicating less delay. AIMS scores range from 0 to 100, with lower scores indicating more delay and higher scores indicating less delay.

### Secondary Outcomes

#### Home Environment, Maternal Practice, and Parent Knowledge

The enhanced developmental intervention group had higher scores on the Interaction Rating Scale subscales for infants’ autonomy, responsiveness, empathy, motor self-regulation, and emotional self-regulation and mothers’ sensitivity, responsiveness, respect to the child autonomy, cognitive growth fostering, and caregiver’s subscale. The enhanced developmental intervention group also had higher scores for the overall Interaction Rating Scale scores that served as a measure of the quality of parent-infant interaction; most effect sizes were moderate (Table 3). No differences were found on measures of development opportunities available at home, parental care practices, or parental knowledge regarding infant development (eTable 2 in Supplement 2).

**Table 3.** Child-Mother IRS Scores by Group at 12 Months of Life^a^

Child-mother IRS outcome	Study group, mean (SD)		Mean difference (95% CI)
	EDI	UC	
Child social skills			
Autonomy	4.1 (1.2)	3.1 (1.5)	1.0 (0.4 to 1.6)
Responsiveness	4.1 (1.4)	3.0 (1.5)	1.1 (0.4 to 1.8)
Empathy	3.7 (1.4)	2.7 (1.5)	1.0 (0.3 to 1.7)
Motor self-regulation	4.7 (1.0)	3.9 (1.4)	0.8 (0.1 to 1.5)
Emotional self-regulation	4.1 (1.2)	3.1 (1.4)	1.0 (0.4 to 1.6)
Child social skills subscale	20.6 (4.9)	15.9 (6.1)	4.7 (3.6 to 5.9)
Mother parenting skills			
Sensitivity	6.4 (0.9)	5.6 (1.6)	0.8 (0.1 to 1.5)
Responsiveness	5.7 (1.8)	4.2 (2.3)	1.5 (0.7 to 2.3)
Respect to child’s autonomy	6.1 (1.4)	5.3 (1.7)	0.8 (0 to 1.6)
Social-emotional growth fostering	6.6 (1.0)	6.1 (0.9)	0.5 (−0.1 to 1.1)
Cognitive growth fostering	5.6 (2.0)	3.9 (2.4)	1.7 (0.8 to 2.6)
Mother parenting skills subscale	30.4 (6.4)	25.3 (7.9)	5.1 (3.6 to 6.6)
Mother-child interaction dyads	51.0 (9.4)	40.9 (12.7)	10.1 (6.9 to 13.2)

Abbreviations: EDI, educational development intervention; IRS, Interaction Rating Scale; UC, usual care.

^a^ IRS scores range from 11 to 55, with lower scores indicating lower competence and higher scores indicating higher competence.

#### Infant Development

With few exceptions, scores on BSID-III cognitive, language, and motor scales and AIMS were higher among infants randomized to enhanced developmental index, compared with the usual care group (Table 2, Table 4, and eTable 3 and eFigure in Supplement 2). eTable 4 in Supplement 2 presents research activities for the enhanced developmental intervention and usual care groups.

**Table 4.** Prevalence and Relative Risk for Delays: EDI and UC

	Prevalence of delays, No. (%)		RR (95% CI)
	EDI group	UC group	
**BSID-III**			
Cognitive scale			
4 mo	9 (18)	13 (26)	0.7 (0.3-1.5)
8 mo	4 (10)	18 (35)	0.3 (0.1-0.7)
12 mo	7 (16)	8 (17)	0.9 (0.4-2.4)
18 mo	5 (10)	8 (15)	0.6 (0.2-2.2)
Language scale			
4 mo	15 (30)	26 (51)	0.6 (0.3-0.9)
8 mo	10 (20)	24 (47)	0.4 (0.2-0.8)
12 mo	8 (16)	20 (39)	0.4 (0.2-0.8)
18 mo	9 (18)	22 (44)	0.4 (0.2-0.9)
Motor scale			
4 mo	13 (27)	17 (34)	0.8 (0.4-1.4)
8 mo	18 (36)	26 (57)	0.6 (0.4-0.9)
12 mo	16 (33)	31 (66)	0.5 (0.3-0.8)
18 mo	8 (18)	20 (41)	0.4 (0.2-0.8)
**AIMS**			
4 mo	22 (45)	30 (61)	0.7 (0.5-1.1)
8 mo	16 (32)	26 (52)	0.6 (0.3-1.0)
12 mo	10 (21)	23 (47)	0.4 (0.2-0.8)
18 mo	3 (5)	21 (44)	0.1 (0-0.7)

Abbreviation: AIMS, Alberta Infant Motor Scale; BSID-III, Bayley Scales of Infant and Toddler Development–Third Edition; EDI, educational development intervention; RR, relative risk; UC, usual care.

## Discussion

In this randomized clinical trial, we evaluated the hypothesis that enhanced developmental support, provided by caregivers, would improve developmental outcomes of infants at risk for developmental impairments due to preterm birth and low family income. The intervention included training provided by a multidisciplinary team of individuals with specialized knowledge of infant development and early intervention, beginning in the NICU and continuing after discharge. This study differs from previous studies of developmental interventions for high-risk infants due to its implementation in an LMIC. The major finding is that infants randomized to enhanced developmental support scored higher on standardized assessments of early cognitive functioning, the primary outcome, at 18 months. In addition, the intervention was associated with higher cognitive scores at earlier ages (4, 8, and 12 months) and with higher scores for language and motor development at 18 months of age and earlier. These findings suggest that primary caregivers were effective facilitators of their infants’ development, and motor interventions are easier for parents to understand and implement.

Of note, the effect of enhanced developmental support was somewhat greater on motor development, compared with cognitive and language development. While this difference in effect size might be explained solely by chance, another explanation is that during infancy, achievement of major motor developmental milestones occurs during the first 18 months, whereas early cognitive and language milestones are achieved at older ages. Another plausible explanation is that interventions used by parents to support motor development, as opposed to cognitive or language development, might be more easily understood and implemented by parents. In addition, parents might experience more gratification from their efforts to enhance motor development, as they watch their infant achieve a series of motor milestones over a relatively short interval.

Previous intervention studies have shown benefits of early intervention for children born preterm, especially in cognitive and motor skills, although few have assessed both domains concurrently.^7,10,17,18^ Recently, the Explorer Baby early intervention program for preterm infants without brain injury^18^ reported that infants who received this intervention showed gains in cognitive and language development during the first 6 months similar to gains exhibited by the active control group, which received neurodevelopmental therapy. Systematic reviews^2,19,20,21,22^ have found evidence of effectiveness of early intervention, although in some studies, no benefit was found for language skills. In the Baby Triple P for Preterm Infants study,^22^ a pragmatic trial to test effectiveness of an intervention for preterm infants in home-based conditions in Queensland, Australia, the intervention increased cognitive and motor skills but did not affect behavior. A meta-analysis of early interventions in developed countries^23^ concluded that interventions implemented by families had a positive impact on cognitive development, an inconsistent impact on motor development, and no effect on language development. The authors pointed out the lack of data on early interventions for children born preterm implemented in LMICs.^23^ Others have noted that the impact of interventions for preterm infants could be increased by training parents in the care of the premature child during the neonatal hospitalization, with continued developmental monitoring and supports for the child and parents after discharge.^19^ The benefit of such an approach in LMICs is suggested by our findings.

Typically, the primary caregivers for high-risk infants, such as those enrolled in our trial, have many opportunities to shape the social and learning environment for their infants, hence differences in children’s development can reflect variations in parent-infant interactions and the home environment.^18,19,24,25^ During infancy, mothers can use specific handling strategies to enhance their infants’ postural control during feeding, bath, play, and sleep.^7,26^ However, these aspects of parents’ care were not enhanced by the intervention we studied.

The enhanced developmental intervention group had higher scores for mother-child interactions. These interactions can be negatively affected by the experience of mothers of a prolonged neonatal hospitalization, potentially leading to depressive symptoms, feelings of powerlessness, lack of confidence in parenting competency, and decreased ability to engage sensitively and emotionally with their infant.^26,27^ Less optimal mother-infant interaction could contribute to infants being less attentive and responsive to their mothers’ social interactions.^19,27^ Conversely, higher quality mother-child interactions promote the child’s responsiveness and empathy to the mother and the motor self-regulation.^28^ The bidirectional nature of the mother-infant interaction involves the infant’s gestures and responses such as smiles, crying, and pointing in response to the mother’s care, thereby eliciting and sustaining even more responsiveness from the mother during routine interactions.^7,10,29^ On the other hand, the mother’s understanding of and reaction to the child’s behaviors facilitate a dyadic relationship that benefits the infant.^23,30^ A premise underlying the intervention that we studied is that strengthening the mother-child interaction provides an enhanced foundation for maternal well-being and infant development.^28^ Other home-based interventions have had similar effects on maternal sensitivity to their preterm child’s needs.^31^

### Strengths and Limitations

This study has several strengths, including the multifaceted continuous intervention that included parent support in the NICU until discharge and in the home and clinic after discharge, use of a randomized clinical trial as a rigorous test of the efficacy of the intervention, and the implementation in an LMIC, a setting where fewer trials have been completed. Another strength is the inclusion of multiple infant developmental domains and mother-infant interaction as outcomes, and evaluation of these outcomes longitudinally for the first 18 postnatal months, allowing for evaluation of developmental trajectories.

A limitation of our study is that follow-up ended at 18 months of corrected age. Since preterm infants are susceptible to adverse outcomes that might not manifest until school age, such as intellectual disability and psychiatric problems, evaluations of long-term outcomes are needed to assess whether intervention programs like the one we studied have sustained effects on neurodevelopment outcomes of preterm children beyond infancy.^20,28^

## Conclusion

In this randomized clinical trial, compared with usual care for very preterm infants, a continuous and multifaceted early intervention for preterm infants, beginning in the NICU and continuing after discharge, and implemented for low-income families, promotes better neurodevelopment outcomes and mother-child interactions. This program is an effective strategy for promoting preterm development in LMICs. If confirmed by other studies, our results suggest that the intervention described herein could improve outcomes for preterm infants in other LMICs.

## Notes

Supplement 1.
Trial Protocol

Supplement 2. **eTable 1**. Neonatal Outcomes for Randomized Participants **eTable 2.** Maternal Practice and Knowledge and Home Opportunities **eTable 3**. Comparison of Cognitive, Language, and Motor Scores for Enhanced Developmental Intervention and Usual Care Groups **eTable 4.** Research Activities for Enhanced Developmental Intervention and Usual Care Groups **eFigure.** Longitudinal Changes: BSDI-III Cognitive, Language, and Motor Composite Scores for EDI and UC Groups

Supplement 3.
Data Sharing Statement
