# Supplementary material for: Parent-Guided Developmental Intervention for Infants With Very Low Birth Weight: A Randomized Clinical Trial
Source: JAMA Netw Open. 2024 Jul 17;7(7):e2421896. doi: 10.1001/jamanetworkopen.2024.21896 (PMC11255911; doi:10.1001/jamanetworkopen.2024.21896)
Supplement: Supplement 1. — Trial Protocol [file jamanetwopen-e2421896-s001.pdf]

Anexo I (versão em inglês)

Submission Date: 08/30/2015

Proposal Information

A. Organization

Organization Name: Hospital de Clinicas de Porto Alegre

Institutional Official authorized to submit and accept grants on behalf of organization:

Prefix	*Prof Dr Rita de Cassia*	Surname	*Silveira*
Title		*Telephone*	5133315726/5198667912
		Fax	51 33289270
Address	*Silva Jardim 1155/ 701.Porto Alegre-RS Brazil*		
E-mail	drarita.c.s@gmail.com		
Web site	rcsilveira@hcpa.edu.br		

B. Project

Project Name: Early Intervention Program for preterm infants and their parents: establishing the impact at 18 months corrected age

Principal Investigator/Project Director:

Prefix	*Prof Dra Rita de Cassia*	Surname	*Silveira*
Title	Professor MD PhD	*Telephone*	5133315726/5198667912
		Fax	51 33289270
Address	*Silva Jardim 1155/ 701.Porto Alegre-RS Brazil*		
E-mail	drarita.c.s@gmail.com		
Web site	rcsilveira@hcpa.edu.br		

Amount Requested ($USD): $ 150000,00

Project Duration (months): 24

Estimated Total Cost of Project ($USD): $ 150000.00

Organization's total revenue for most recent audited financial year ($USD): $

Application Table of Contents

I. Main Goal ____ 4
II. Executive Summary ____ 4
III. Context ____ 7
IV. Project Framework ____ 8
V. Sustainability ____ 10
VI. Organizational Capacity and Management Capability ____ 10
VII. Citations ____ 11
VIII. Appendices ____ 12
- A. Budget Spreadsheet ____ 12
- B. Budget Narrative ____ 13
- C. Biographical Information ____ 15

I. Main Goal

To develop a program of early intervention for very preterm infants that allows families to apply it continuously at home.An additional objective of this research is to quantify the results of early stimulation on improvement of cognition and motor skills.

II. Executive Summary

Introduction- Born prematurely (soon to born) and its consequences cause major impact on society and health indicators of population. According to the 2012 ''Born Too Soon: The Global Action Report on Preterm Birth'' of the World Health Organization, Brazil is ranked 10th among the countries with the highest number of preterm live births and 16th in deaths due to complications of prematurity [1]. The data from 2012 indicate that approximately 3 million babies are born in Brazil each year, 350 000 of whom are born with less than 37 weeks of gestation, and born prematurity index is higher in the last three years, including preterm very low birth weight infants (gestational age less than 32 weeks and birth weight less than 1500 grams). More than half of the preterm infants with birth weight less than 1500 grams born in public university centers of Brazilian Neonatal network Research at a gestational age GA of 23–33 weeks died or were discharged with severe pulmonary, neurological or ophthalmological complications[1,2].

From the neonatal point of view, assistance in the delivery room with effective resuscitation is important and among the survivors is imperatives a continuous follow up clinic program after the hospital discharge.Follow up program after discharge is a continuous of neonatal and perinatal care, and should provide conditions to monitor growth, development and common morbidities with a multidisciplinary team able to fully assess the child and the caregivers, parents, all family and school. Early intervention for high risk preterm infant must focus in the parents-infant relationship, environment and behaviora attitudes [3]. It is possible that a care process modified by households is beneficial for neurodevelopment premature, both in the cognitive aspect as motor.

Background

Preterm infants are high risk for delayed neurodevelopment. There are several intervention programs attempting to improve their outcome**.** According Cochrane Review there is a great deal of heterogeneity between studies due to the variety of early intervention programmes and gestational ages included in these programmes [3] .

Early intervention programs for preterm infants that focus on development while the babies are still in the hospital and post discharge from the hospital, and into the community setting may have a greater impact on long-term morbidity as they are able to focus more on family factors and the home environment. Interventions that are aimed at enhancing the parent-infant relationship focus on sensitizing the families to infants cues and teach appropriate and timely response to the preterm infant's needs, possibly that early high-quality parent-infant or mother-infant interactions positively influence cognitive and social development in children [3,4]. **Despite of those evidences, the role of the family applying those programs at home is not well studied especially in social deprived environments**

Executive narrative project

There are several intervention programs involving multisensory and motor stimulations such as, gym, auditory, visual, vestibular and tactile stimulations. We propose to study a continuous program of early intervention involving very preterm infants' families in their first 12 months of life taking the chance of their neuronal plasticity during this period. Preterm infants born in our institution will be included when they complete 48 hours after birth (first intervention). All preterm infants and their mothers will be followed during neonatal period and pre hospital discharge they will have a second intervention (to measure parental bond). After discharge they will be conducted to follow up program and we will divided all very low birth weight infants included in the study in two groups according previous randomization:

1. Standard care with motor, and cognition evaluation and intervention according to their needs
2. Program of early intervention with parents' orientation independently of f the standard evaluation and care that will be performed.

All phases of neurodevelopment will be evaluated, and the parents will be oriented to stimulate motor, language and cognition iteratively and continuously at home; this is a innovative method to improve very preterm neurodevelopment outcome.

Randomization: in neonatal period, preterm infants will be sequentially randomized when they completed 48 hours after birth in:

Group 1- conventional group (CG): standard care, according to the routine care of the NICU (skin-to skin care by mother, kangaroo care).

Group 2- intervention group (IG): skin-to skin care by mother (kangaroo care) plus massage therapy by mothers. They will receive the tactile-kinesthetic stimulation by mothers from randomization until hospital discharge. Intervention performed exclusively by the mothers was based on studies regarding the application of skin stimulations and passive exercises in preterm infants[5-7].

Both groups will receive skin-to skin care by mother (Kangaroo Care) according to the routine care of the NICU, In previous study, we performed the same intervention as follows[8]:

The mothers will be instructed to perform one stage at a time, and will be supposed to end the sequence within 15 min, four times per day with an interval of 6 hours. The tactile stimulation will be performed on the

cutaneous surface and the muscular area corresponding to the temporal, frontal, periorbital, nasal and perilabial regions of the face; the external side of the upper and lower limbs; and the soft parts of two or three fingers grouped will be passed gently with moderate pressure, up to three times in one direction and three times in the opposite direction. The kinesthetic stimulation will consist of passive exercises (flexion and extension) of upper and lower limbs, one limb at a time and up to three times at each articulation (wrist, elbow, ankle and knee); one of the hands supporting the stimulated limb and the other hand performing the movements[8].

To ensure a safe application of the intervention, mothers of the IG will be instructed to observe the newborns' tolerance signs, avoid excessive stimulations, keep the babies lying on their back, with stretched limbs close to the body, and perform the kinesthetic stimulation to one limb at a time. Researchers of our team will have regular meetings with mothers included in IG every 48 hours to assure that they are doing the intervention as instructed and to check the parental bond[9].

In a previous publication we demonstrated that massage therapy by mothers combined to skin-to-skin care during neonatal hospital stay improved neurodevelopment outcome at 2 years corrected age[10] .

We will evaluate parental stress and parental infant bonding at hospital discharge in all preterm infants included in the study and survival in the neonatal period, will be the second intervention moment .

PARENTAL BONDING INSTRUMENT (PBI) will be applied by a professional blind to the group to which the child belong.

The following neonatal and sociodemographic data will be collected for between-group comparisons: parental ages maternal educational achievement was measured in years of formal schooling, as reported in questionnaires administered to mothers during follow-up visits; gestational age; birth weight; weight at discharge or at the 40th week of postmenstrual age (whichever came first) and at 12, 18 24 months (corrected age); adequacy of weight for gestational age at birth and for age at discharge or 40th week of postmenstrual age (whichever came first); number of infants with head circumference below 10th percentile at birth and at discharge or at the 40th week of postmenstrual age (whichever came first); growth velocity from birth until discharge or at the 40th week of postmenstrual age (whichever came first); presence of early or late-onset sepsis,necrotizing enterocolitis (NEC); respiratory distress syndrome (RDS), periventricular/intraventricular hemorrhage (PIVH, grades II to IV)), and Cystic leukomalacia and noncystic periventricular leukomalacia, bronchopulmonary dysplasia,;retinopathy of prematurity (ROP). Gestational age will be determined on the basis of maternal data and confirmed by early ultrasonography and by the neonate's initial physical examination.

During the follow up program the research group will determine the number of infants with weight, length, and head circumference less than 2 standard deviations of z-score (World Health Organization growth curves) at 12 and 18 months' corrected age.

All preterm VLBW infants (birth weight <1500 g) born at the Hospital de Clínicas de Porto Alegre (HCPA) are routinely referred to the Neonatology outpatient clinic for monthly follow-up visits until 6 months of corrected age (CA) , bimonthly from 7 up to 12 months corrected age, and every 3 months thereafter until age 24 months, according to routine hospital practice. According randomization group 1 will have standard care of a traditional follow up clinic taking care of the demands according to their necessity and the group 2 will receive orientation for a continuous global simulation at home besides the usual appointments to the follow up clinic, monthly in the first semester corrected age and bimonthly in the second semester until 12 months corrected age and every 3 months thereafter until age 24 months corrected age.

Follow up appointments, home visits and intervention during follow up program

The systematic orientation program for early intervention will be according to developmental milestones, anticipating in a month evolutionary step acquisition of motor and / or cognitive expected for corrected age. In the first half of corrected age, the mother, father and / or corresponding caregivers receive simple guidelines to encourage large motor skills, fine and cognition.

Up to three months of corrected age: stimulation in the position lying in bed.

Gross and fine motor stimulation: Crossing arms and relaxation movements (play in the bath for 5 to 10 minutes, beating hands and feet in water, rubber animal). Gymnastic movements (flexion and passive extension of the upper and lower limbs) with the purpose of adopting child body awareness.

Cognitive stimulation: Getting close and speak slowly, singing low. Mobile to look up, black and white gloves to put in the hands' mother to play and the child turn your head 180◦C.

Between 3 and 6 months of corrected age: Gross and fine motor stimulation: the child lie on his back for a big plastic ball holding her thighs. Bending light and the ball slowly forward and backward, side to side, in order to prepare for the sitting position and obtain equilibrium. Lying on her back with a mobile in the belly height, arms outstretched pad and mother holds the child's hands, showing her that you should try to catch with their feet, learning to ride. Teach touch objects with different textures (soft, hard, rough, rough) describing the characteristic touch (along stimulates cognition).Cognitive stimulation: listen to music, singing and reading simple words and short sentences. After bathing, and while dry with the towel body parts, the mother says: the foot, the hand, arm and so on, speaking slowly with the child. In the mirror, show and nominate: eyes, mouth, nose of the child and the mother, for the same become aware of their individuality.

From six to 12 months corrected age: Gross and fine motor stimulation: gymnastics intends to tone the muscles in order to prepare for the first sitting position and then standing without support, to walk independent. Using a large plastic ball (the ball should not be too full) the mother is oriented to hold the child against him, one hand holding his knees thereof and the other the chest. Tilt the child's chest towards the ball until it rests in the hands

and make a slight effort to rise. In the sequence of months, run the ball, causing the child release both hands. It is important to advise the mother to release the hip child gradually.
Use the floor with EVA material for the child to have displacement space.
To stimulate fine motor, is guided to offer magazines to be torn by the child, showing how to do it quietly. Once torn into several pieces, teach to make a paper ball with the pieces and play ball with the child.
The exercises of time should not exceed 15 minutes and must seem games in the mandatory frequency of three times / week.
Cognitive stimulation: Black and white balls; and the later; colored balls in the same place for the child find it easier. Talk repeatedly reinforcing each child's achievement. From 8 months of corrected age, display and nominate the body parts: head, belly, hand, foot, mouth, nose, eyes. Read books and show the animal pictures, repeating the name of each figure well paused and with the mouth wide open.
The time of these activities must not exceed 15 minutes and must seem game.Three times / week (alternating with gross and fine motor stimulation).
We will be perform two orientations every three months for cognitive stimulation, fine and gross motor , totaling the 10 home visits promoting guidance and supervision sessions. Systematic orientations will be delivered to parents in all medical appointments at follow up clinic.
There will be home visits in order to evaluate the comprehension of the orientation and to be sure that the intervention has been done by families.
There will be a multidisciplinary team involved in the whole study and we will have a critical view of the intervention impact (final evaluation) in both; conventional and intervention groups.
The infants will be evaluated in relation to their motor, and cognitive neurodevelopment using AIMS and Bayley III scales between 12 and 18 months corrected age.
AIMS (Alberta Motor Infant Scale): a blinded physiotherapist will evaluate the children of both groups between 12 and 18 months with Alberta Infant Motor (AIMS) scale in all eligible patients.

A global evaluation will be performed at one year chronological age with Bayley and AIMS scales.

The evaluation will be conducted in the presence of parents or caregivers in a safe surface with room for the child move around during the evaluation. The examiner will interact with the child to encourage response, but physical facilitation of movement should be avoided. During the evaluation, they are punctuated behaviors more or less mature within the motor repertoire of the child in each position (supine, prone, sitting and standing). This repertoire is called "motor" window. All items priced within the window motor and the window motor to the previous items are scored. The evaluation of the end, the child will receive a score based on the sum of the items scored on each posture, called raw score. This score will be observed in a standardized chart to find the baby development percentile according to the chronological age or corrected. Percentiles instrument standards are: 5%, 10%, 25%, 50%, 75%. According to this percentile baby's development can be classified into three categories: normal or typical (percentile> 25%), suspicious (percentile> 5% and ≤25%), abnormal or atypical (percentil≤5%) [11]
BSDI-III: *Bayley Scales of Infant and Toddler Development* third edition: The Bayley Scales of Infant and Toddler Development, Third Edition, will be used for assessment of neurodevelopment at 12 and 18 months' corrected age. The scales will be administered at the hospital clinic, on the same day of each follow-up visit, by a psychologist who was blinded to group allocation. Cognitive, motor, and language development will be considered normal if higher than 89; below average if 80 to 89; borderline if 70 to 79; and extremely low if less or equal 69. Examine all the facets of a young child's development according manual[12].
AIMS and Bayley Scales are recommended to use together and a different ages because false positives are common and therefore it is beneficial to follow-up children at high risk of motor impairment at more than one time point, or to use a combination of assessment tools[13].

Patient of both groups will be evaluated for parental infant bonding.PARENTAL BONDING INSTRUMENT (PBI) : PBI is a self-administered Likert scale (0 to 3) instrument, with 25 questions related to father and mother, in which subjects answer how similar those behaviors were to their parents' behavior until the age of 16 years. The instrument measures two constructs: the first one is affection, which is more consistent and clearly bipolar (affection, heat, availability, care, sensitiveness versus coldness and rejection); the second construct is control or protection (control, intrusion versus encouragement of autonomy).
The prevalence rates of exclusive breastfeeding and mixed feeding at 6 months corrected age will be recorded, to measure maternal bond, in both groups.

If this program shows a good result it can be expanded for the whole preterm population in order to improve their neurodevelopment outcome.

III. Context

Developing countries must allocate their resources according to their conditions. Preterm infants require several different professionals to take care of them, and many places do not have enough people to take care of all preterm infants. Our decision was based in a critical cut off point of gestational age and birth weight according different studies[14-17]. We decided to approach our study in a group of preterm infants with gestational age less

than 32 weeks or those with birth weight less than 1500 grams independently of their gestational age. The follow up clinic of our hospital take care of preterm infants with gestational age less than 32 weeks or those with birth weight less than 1500 grams independently of their gestational age cared at our NICU evaluating periodically their neurodevelopment and forwarding to specialized professionals according to their necessity.

The multidisciplinary approach to early interventions may result in better performance and quality of life in the future for these children .Preterm infants are susceptible to several handicaps like neurological injuries, growth failure, psychiatric problems, visual and hearing deficits, fine and gross motor problems and language problems. Early systematic intervention is a context of the research that will be conducted, independently of formal enriched environments, according previous publication[10]. A multidisciplinary team is required to form a follow up clinic[14-17]. This clinic must be coordinated by a neonatologist that understands the infant as a whole.

The program team is as follows:

Member of the team	Role in the team
Pediatrician/Neonatologist	Coordination ,evaluate growth and screening of development, take care of the general clinical medical problems
Psychologist	Evaluate neurodevelopment using scales, psychological problems, parental infant bonding
Pediatric Neurologist	Manage seizures, cerebral palsy, swallowing problems
Ophthalmologist	Evaluation of ROP, visual acuity, strabismus
Ear, nose and throat doctor	Evaluation and management of hearing problems
Nutritionist	Management of growth failure
Speech Therapist	Speech problems and swallowing problems
Nurse	Immunization and Hygiene control
Team for home visits	Pediatrician, neuropsychologist, speech therapist, physiotherapist, nutritionist, nurse, occupational therapist

Our main hypotheses is that a continuous global early stimulation done by parents at home for very preterm infants is better than the traditional one, and it can be offered to many very preterm infants even in poor environments.

Many studies have been focused in motor development after early intervention [18]. Recent neuroplasticity literature suggests that intensive, task-specific intervention ought to commence as early as possible and in an enriched environment, during the critical period of neural development. Active motor interventions are effective in some populations, however the effects of those active motor interventions on the motor outcomes of infants with CP have been researched only in a pilot study[19]. Goals - Activity - Motor Enrichment): protocol GAME, was used in that pilot study[20] Cognition is very poor evaluated after early intervention programs. Recently, the effects of the Teach-Model-Coach-Review instructional approach on caregivers' use of four enhanced milieu teaching (EMT) language support strategies and on their children's use of expressive language were examined and the results were positive, but preliminary evaluated[21].

As the outpatient follow-up is an extension of perinatal care, we will monitor the child and his family since during neonatal hospital stay, near the hospital discharge will be checked the parental bond.[,9,14,17].
We are proposing to randomize our very preterm infants in two groups. One group will receive the standard care of a traditional NICU program (kangaroo care) and a follow up clinic taking care of the demands according to their necessity. The other group will receive orientation for the tactile-kinesthetic stimulation by mothers from 48 hours after birth until hospital discharge and a continuous global simulation at home besides the usual appointments to the follow up clinic. Home visitors will go 10 times during the study to their homes to be sure that they are performing the right stimulation.
A blinded physiotherapist will evaluate the children of both groups between 12 and 18 months with Alberta Infant Motor (AIMS) scale.

A blinded psychologist will evaluate neurodevelopment of the children of both groups between 12 and 18 months using Bayley III scale.

Patient of both groups will be evaluated for parental infant bonding.

The project will be developed by Dr Rita C Silveira and Dr Renaro S Procianoy in colaboation with Dr T. Michael O'Shea, MD, MPH **.** Renato S Procianoy MD PhD and Rita C Silveira MD PhD are principal investigators These researchers have many previous studies published where the relationship between preterm morbidities and neurodevelopment outcomes have been shown. Dr O'Shea has extensive experience with studies conducted by NICHD and is currently one of the coordinators of the study group Elgan (Extremely Low Gestational Age Newborns) supported by NICHD .

IV Project Framework

Project Framework Table

	Results	Period of Activity
**Objective 1:**<br>To implement a program of continuous and global intervention for preterm infants to be delivered by their families	Improve interaction parental infants since neonatal period with massage therapy by mothers<br>Decrease parental stress<br>Improve neurodevelopment at 18 months corrected age	**During the whole study period**.<br>It will start one month after receive the grant. There will be meetings with the team. All the team will need to be trained to teach the tactile-kinesthetic stimulation by mothers<br>The patients will be allocated to the study up to a total of **100 (50 in each group)** and sequentially randomized when the patients complete 48 hours after birth. We will exclude congenital malformations and parents'refusing to participate in the study.<br>At the end professionals with training to carry out the testings for development assessments will provide the results.
**Objective 2:***<br>Advise and improve the skills of care givers in respect to children's needs	Reduce to the minimum the lost for follow up (home visits)<br>Decrease parental stress that will be measured previously<br>Improve parental infant bonding that will be measured in the beginning<br>Home visits to be sure that the interventions are performed	Patients will be randomized to two groups. At admission of the study the intervention will promote a moment of care and further interaction through massage therapy performed by mothers . Following consist of guidelines and measures to promote early intervention with parents' orientation independently of f the standard evaluation and care that will be performed. There will be daily sessions of 10 to 5 minutes each one.
**Objective 3:**<br>Evaluate the impact of the intervention in the neurodevelopment of the children	More strengthened ties to the program start , as measured by PBI resulting in higher scores of attention, care and protection .<br>Number of patients with the Bayley III scale normal for corrected age<br>Number of patients with AIMS scale normal for corrected age.<br>Statistical measures of the differences between the groups (intervention and conventional approaches) .	At discharge of neonatal unit (PBI) we will have the first evaluation. After 10 sessions and home visits we will have the follow up evaluations.<br>AIMS and Bayley III Scales at 12 to 18 months corrected age<br>A global neurodevelopmental evaluation will be obtained at one year chronological age..

Project Framework Narrative

Very low birth weight preterm infants that were born in the Hospital de Clinicas de Porto Alegre 48 hours after birth, without any exclusion criteria will eligible for the study. So, the first activity will be to obtain written informed consent from the parents.**Randomization will be performed by research who will not be responsible for any intervention, nor outcome measures of parental bond, motor and cognitive outcomes.** The same researcher will register all neonatal data, discharge variables and the follow-up data, during the appointments. The allocation of the subjects will be maintained until the complete sample size calculation based on the number of premature infants who survive the equivalent term or hospital discharge, routinely being candidates for outpatient follow-up clinic.

A trained research will evaluate parental stress and parental infant bonding at hospital discharge in all preterm infants included in the study and survival in the neonatal period. PARENTAL BONDING INSTRUMENT (PBI) will be applied by a professional blind to the group to which the child belong.

Activities

Multidisciplinary team prepares eligible patients for hospital discharge and follow up program, promotes regular meeting with the mothers, and high standard guidance for all patients, followed by two groups of care according to the previous randomization into two arms :1. Standard care with motor, and cognition evaluation and intervention according to their needs.2. Program of early intervention with parents' orientation independently of f the standard evaluation and care that will be performed.

In the initial stage parental bond will be evaluated and worked to stimulate greater attachment.

Three pediatrician/neonatologist will be evaluating monthly, until 6 months of corrected age (CA) , bimonthly from 7 up to 12 months corrected age, and every 3 months thereafter until age 24 months, according to routine hospital practice, all very low birth weight infants.. According randomization group 1 will have standard care of a traditional follow up clinic taking care of the demands according to their necessity and the group 2, will receive orientation for a continuous global simulation at home besides the usual appointments to the follow up clinic, monthly in the first semester corrected age and bimonthly in the second semester until 12 months corrected age and every 3 months thereafter until age 24 months corrected age. Multidisciplinary team will participate of these activities all the time.

The teaching activities and educational programs for the families will be the focus of our research group throughout the study..

V. Sustainability

We will provide conditions to establish an early stimulation protocol according to corrected age exercised by the family.Visual, auditory, gross and fine motor skills, socialization, definition and body parts knowledge will be worked out as previously described in this project

Results/Critical Milestones

The major result that will be used to measure progress is related to main goal: to implement a program of early intervention for very preterm infants that allows families to apply it continuously at home. Additionally, to quantify the results of early stimulation on improvement of cognition and motor skills.
For a critical evaluation, outcomes measures of interest will be those that assessed progress in motor and cognitive skill acquisition at any time after early intervention, provided that score reaches 20 % higher.
Sample size calculation: the sample size is calculated on the basis of the results of many studies that assessed improvement of motor and mental development or cognitive or language acquisitions. All these studies obtained minimum scores 20% higher after early intervention. For a 5% level of significance and a statistical power of 80%, a sample size of 84 patients will be required to detect a 3-point between-group difference in development scores [10,19,20,21]. The allocation of the subjects will be maintained until the complete calculated sample size based on the number of premature infants who survive until hospital discharge. A number of 20% (16) will be added, considering possible loss or death during the follow-up .<u>A total sample size will be 100 preterm infants</u>
At the end of the study we expect that the group that had a continuous and global early intervention presents a better neurodevelopment than the group that received the standard and traditional care.
If we can show that a continuous and global early intervention at home performed by low income families is better than the standard care for very preterm infants, this kind of program may be applied elsewhere in the world.
Results Measurement
Monitoring the progress of this research is fundamental for assuring that all activities will be achieving stated milestones. The selection of adequate method for generating the randomization sequence is important to results measurement.
All research team need to offer opportunity for motor learning and "training," for the purposes of this research we considered training inclusive of environmental adaptations to enhance training, as form of motor-specific

enrichment. An important result will be to enhance parent-infant interaction, educate parents about assisting their child's skill development, provide opportunities for active motor learning (self-generated motor activity). Parental bonding instrument (PBI) will do the measurement of this result.
The outcomes will be measured by Bayley III Scale, Alberta Infant Motor Scale are quantitative.
Statistical Analysis: quantitative variables will be expressed as means and standard deviations, when symmetrically distributed or as medians and interquartile ranges otherwise. T Student or Mann Whitney tests will be use for comparisons between the groups.Qualitative variables will be expressed as absolute and relative frequencies. Pearson chi-square test will be employed to determine the association between categorical variables, with adjusted residuals in case of statistical significance.
All the people that evaluate the patients will be blinded to intervention.

VI Organizational Capacity and Management Capability

A. Organizational Capacity and Facilities:
The study will be developed at Hospital de Clínicas de Porto Alegre. Hospital de Clinicas de Porto Alegre has more than 4000 deliveries /year and the NBICU has 120 to 150 very preterm infants admitted yearly. Obstetric Unit of the hospital has 150 deliveries with gestational age ≤ 32 weeks per year. There is a 20 bed level III Neonatal Intensive Care Unit with conventional and high frequency ventilation, nitric oxide therapy, bed cranial and cardiac echocardiography available any time during the day, 3 on call board certified Neonatologists the whole day. There is also a team available for birth assistance anytime during the day.
There is a follow up clinic that follows all the preterm infants less than 32 weeks gestational age or less than 1500 grams birth weight independently of gestational age.

B. Management and Staffing Plan for this Project:

1. Management and planning of the study is in charge of the principal investigators.

There will be:

Neonatologists (3) for the medical appointments,

Psychologists (3) (one for Bayley III scale and 2 for parents' orientation of the intervention),

Physiotherapists (3) (one for AIMS and 2 for motor stimulus),

Nurses (2) ,for intervention in the NICU

Nurses (2), orientation and home visits during the follow up

Speech therapists (3) for oral stimulation, speech testing and speech stimulation,

Ocupational therapist (1) for intervention and.

Two researchers student to collect data and data bank .

The multidisciplinary team for home visits: Pediatricians, neonatologists, nurses, speech therapist, neuropsychologist, physiotherapist, nutritionist and occupational therapist.

Management and Staffing Plan for this Project
Principal Investigators:
Rita C Silveira. Associate Professor of Pediatrics and Neonatology, Universidade Federal do Rio Grande do Sul, Head of the Neonatal Follow up Program of Hospital de Clinicas de Porto Alegre (HCPA) with large experience in follow up program for over ten years. Head of Neonatal Section of HCPA .She has more than 65 papers indexed in PubMed and she is PQ2 of CNPQ.
Renato S Procianoy Full Professor of Pediatrics and Neonatology, Universidade Federal do Rio Grande do Sul, Head of Research Center of Hospital de Clinicas de Porto Alegre with large experience in neonatal care and clinical research. He has more than 120 papers published indexed in PubMed and he is PQ1C CNPQ.
Co-Investigator (collaboration)
T. Michael O'Shea, MD, MPH: Professor of Pediatrics, University School of Medicine North Caroline at Chapel Hill , Head Neonatal Perinatal Medicin Unit, North Caroline. USA. Elgan -2 study coordinator linked to the American Research Network with support from NICHD . More than 300 publications on Pubmed . Professor O'Shea will help with the evaluation of the program results , and analyze critically if the early intervention program conducted by parents in Brazil is feasible to be applied to other places in the world with low socio economic conditions.

VII. Citations

Procianoy RS, Mendes EW, Silveira RC. Massage therapy improves neurodevelopment outcome at two years corrected age for very low birth weight infants. Early Hum Dev. 2010; 86 (1):7-11.

Silveira RC, Procianoy RS. High plasma cytokine levels, white matter injury and neurodevelopment of high risk preterm infants: assessment at two years. Early Hum Dev. 2011 Jun;87(6):433-7.

Silveira RC, Fortes Filho JB, Procianoy RS. Assessment of the contribution of cytokine plasma levels to detect retinopathy of prematurity in very low birth weight infants. Invest Ophthalmol Vis Sci. 2011 Mar 10;52(3):1297-301.

Oliveira MG, Silveira RC, Procianoy RS .Growth of very low birth weight infants at 12 months corrected age in southern Brazil. J Trop Pediatr. 2008 ;54(1):36-42.

Filipouski GR, Silveira RC, Procianoy RS. Gestational Age on Neurodevelopment of Very Low-Birth-Weight

Preterm Infants Am J Perinatol 2013;30:673–680.

Procianoy RS, KochMS, Silveira RC. Neurodevelopmental outcome of appropriate and small for gestational age very low birth weight infants. J Child Neurol 2009;24: 788–794.

T. Michael O'Shea, L. Corbin Downey, Karl K. C. Kuban Extreme prematurity and attention deficit: epidemiology and prevention Front Hum Neurosci 2013; 7: 578.

Seetha Shankaran, Aiping Lin, Jill Maller-Kesselman, Heping Zhang, T. Michael O'Shea, Henrietta S. Bada, Jeffrey R. Kaiser, Richard P. Lifton, Charles R. Bauer, Laura R. MentMaternal race, demography and health care disparities impact risk for IVH in preterm neonates J Pediatr. Author manuscript; available in PMC 2015 May 1.

Silveira RC, Filipouski GR, Goldstein DJ, O'Shea TM, Procianoy RS Agreement between Bayley Scales second and third edition assessments of very low-birth-weight infants. Arch Pediatr Adolesc Med. 2012; 166(11):1075-6.

2. Overview of decision making plans and processes:

Once established our fundamental purpose that is an early intervention programs for preterm infants that focus on development while the babies are still in the hospital and post discharge from the hospital, and into the community setting . promoting a greater impact on long-term morbidity as they are able to focus more on family factors and the home environment.

If we can show that a continuous and global early intervention at home performed by low income families is better than the standard care for very preterm infants, this kind of program may be applied elsewhere in the world.

VIII. Appendices

A. Budget Spreadsheet

	**Year 1**	**Year 2**
**Psychologists**	**R$32480.00**	**R$ 32200.00**
**Speech therapists**	**R$411.00**	**R$ 15600.00**
**Physiotherapists**	**R$14000.00**	**R$23800.00**
**Nutritionist**	**-**	**R$21000,00**
**Travel for presentation in International Meetings for two investigators plus registration and daily charges**	**R$39000.00**	**R$39000.00**
**Nurse/occupational therapists**	**R$ 25000.00**	**R$ 21.000.00**
**Home visits**	**R$32340,00**	**R$ 8400.00**
**Equipment for AIMS e Bayley III scales**	R$14589.00	**-**
**Computers (2)**	**R$ 6000.00**	
**Early intervention instruments**	**R$ 13540.00**	**-**
**Team Approach**	**R$ 1200.00**	**-**
**Meeting's Training**	**R$ 38000.00**	**-**
**Graphic material for stimulation**	**R$4788.00**	**-**
**Schollarship**	**R$39600.00**	**R$39600,00**
**Overhead**		**R$25000.00**
**Estimated cost by year**	**R$ 260,948.00**	**R$ 225,600.00**
**Research's Total cost**	________	**= R$486548,00**

B. Budget Narrative

The budget is basically personnel expenses in order to develop the proposed program.

The cost of the project basically involves human resource multidisciplinary order to perform the intervention in hospital and outpatient before discharge from the NICU (training, training meetings for research) and guarantee

of the multidisciplinary team in follow-up visits (1 randomization arm) and this standard care plus a systematic orientation program for family members to carry out stimuli in the household (arm 2).
Inaddition to the costs of the professionals with the outcome measures. The principal investigators (PI) will be present in these stages of the research both; in the neonatal hospitalization (RSP) and in outpatient follow-up clinic and research programs (RCS).
Training teams for interventions: are twenty meetings in modules of guidelines for the entire multidisciplinary team. Room rentals (35 seats) with audio visual equipment worth R $ 1,900.00 / meeting. Total estimated R $ 38,000.00
Home visits The cost of home visits involve transport of the entire team that will make the monitoring of caregivers at intervals between visits, totaling 10 visits / patient.
It is estimated a total of 420 home visits (according to sample calculation) with shipping mutidisciplinar team in catchment area of 60 km from the center of assistance to R $ 32.00 and R $ 45.00 one way return.
Total transportation to home visits = R $ 32,340.00 in the first year
Total transportation to home visits R = $ 8400.00, in the second year
Training's team in neonatology at randomization, data collection and application of the assessment questionnaire (BDI); achieved by neonatology (3). The researchers will do the guidance of scientific initiation grantees and all research team.
Scholarship (ATP) R $ 550.00 / month by market (6 fellows AT / year): R $ = 39,600.00 in the first year and R$ 39,600.00 in the second year.
Neonatologists: Research teams in neonatology and in outpatient follow-up, International researchers will present partial and final results at international congresses: R $ 39,000.00 / year.
Physiotherapists: make orientation sessions to mothers at all times of interventions following (10 sessions / subject of GI, totaling 420 systematic guidelines following, being 280 the first year and 140 in the second year) .Total R $ 14,000.00 / year 1 and R $ 7,000.00 / year 2.
The total cost for AIMS scale applications by trained physiotherapists is estimated at a total R $ 16,800.00; for both groups (CG and IG), and should be blinded to which group the subject belongs. Total cost of physiotherapy assistance will be R $ 37,800.00.
Nursing / Occupational Therapist: evaluation of maternal care, encourage breastfeeding, kangaroo care are part of the care management. Nurses research that will guide / oversee mothers daily from 48 hours of life, randomized to GI and clinical meetings will receive R $ 18,000.00 in the first year. Occupational therapy that will guide some passive exercises will receive R $ 7,000.00 the first year. These values include all allocated subjects in the intervention group to the time of hospital discharge. Total cost of this activity: R $ 25,000.00.
Following, will participate in the multidisciplinary group with guidelines for improving the care and supervision of early stimulation performed by families in 10 interventions and the follow-up visits. The occupational therapist will do a series of exercises motors in home visits. The estimated cost for this activity is nursing (2) R $ 18,000.00 and occupational therapist (1) R $ 3,000.00.
Psychologist : three in number, one for performing the tests, and ratings neurodevelopmental and two guidelines to make psychoaffective interventions, and behavioral cognitvas parents; preparing mothers for hospital discharge, supervision and evaluations of parental ties, training in a multidisciplinary team. The value of each orientation package in Neonatology will be R $ 4,200.00 / 42 subjects in the intervention group for each professional. Reviews bonds (PBI) will be R $ 120.00 / subject of both groups. R $ 10,080.00. Total cost during hospitalization = R $ 18,480.00. Will guide the mothers at all times of interventions following (10 sessions / subject of GI, totaling 420 systematic guidelines following, being 280 the first year and 140 in the second year) .Total R $ 14,000.00 / year 1 and R$ 7000.00 / year 2.
The total cost for applications of the Bayley scales, congnitiom, language and motor, by qualified and trained psychologist is an estimated total of R $ 25,200.00 / 84 subjects.
Speech therapist : monitoring in care routine in neonatology, with two orientations (one each semester to research group) in the total cost of R $ 411.00. In the outpatient follow-up will be part of the multidisciplinary team that will guide for stimulation and for home visits in the intervention group, performing early stimulation on the axis of language. The estimated cost for this activity is R $ 15,600.00
The cost of materials for stimulation of the child and the testing, professional and applies its own test kit will be detailed below:
Materials for stimulation:

Colored mobile total = R $ 1,260.00
Rubber animals three for each subject included in the Intervention total: R $ 504.00
Colorful rattle without light and with light
total = R $ 2,730.00
Plastic large ball
total R $ 420.00
Black and white woolen gloves , one for each mother / parent intervention group
Total = R $ 210.00
Small colored balls of non-toxic rubber
R$ 3.50 (unit). = Total: R $ 588.00

Small mirror, 5.00 (unit).. Total; R $ 210.00
Books with drawings of animals and objects ,
R $ 18.00 (unit), Total = R $ 1,512.00
Books with stories R $ 15.00 (unit), Total = R $ 1,260.00
EVA Material 1 mx 1 m,
R$ 10.00 (unit) .Total = R $ 420.00
Rag doll, R $ 24.00, Total = R $ 1,080.00
Toy fit, colored (cubes, geoméricas forms) , one for each subject included in Group Intervention
Total = R $ 2,260.00
Wooden poles with 60 cm in size each , two for each subject in the intervention group
Total = R $ 1,050.00
Total general stimulation for materials: R $ 13,504.00

Printed Graphic material

Graphic material and computing for typing of hospitalization data and tracking and guidance for parents (informative brochures), totaling 420 folders (stipulated 10 per visit, 2 colored / time)
Folder R$ 1.00 (black and white) and R $ 3.00 (colored). 8 black and white will be delivered and two color for each subject included in the GI at a cost of R $ 14.00 / patient. Total = R $ 588.00.
Printing Kit growth curves (84), three curves in each group; conventional and intervention
Each kit costs R$ 6.00 / unit (252 kits) Total = R$ 1512.00
Printed kit AIMS Scale (84), an application for each group; conventional and intervention
Each kit costs R$ 12.00 / unit. Total = R$ 1008.00
Questionnaire printed PBI), two applications in each group; conventional and intervention, including tutorial sheets and scores.
Total R $ 1,680.00
Total of printed graphic material R $ 4,788.00

Database and statistical analysis
= R $ 13,200.00 (scientific initiation grantees) in the second year

Nutritional Evaluation
In neonatology, the nutritional management will be performed by the staff of the nutrition service with no cost. The growth velocity will be registered in the reference curves, using the two computers with an appropriate statistical program for nutritional assessment (ANTHRO) .
Following, there will be Nutritionist multidisciplinary group that will make home visits with the team and two nutritionists to perform the growth evaluations in the exact moments in each group (blinded to which group the child was previously allocated).
.
Total nutritional assessment R $ 21,000.00

AIMS DVD (purchase order must be on institutional letterhead or an institutional form) U$ 140,00 and ship U$ 10,00; total U$ 150,00 (R$= 468,00, exchange in 05/25/2015). Package of 50 score sheets U$ 95.90 / UNIT(R$ 300,00). We will need at least four packages (R$1200,00).Total cost R$=1668,00.

BAYLEY–III: Comprehensive Kit in Rolling Case with Scoring Assistant. Includes Administration Manual, Technical Manual, 25 Cognitive/Language/Motor Combination Record Forms, Stimulus Book, Picture Book, Complete Manipulative Set, 25 Social-Emotional/Adaptive Behaviour Questionnaires, 25

Caregiver Report Forms, Fundamental Administration DVD, Observational Checklist, rolling case, PDA Administrative Assistant (Electronic Record From and Scoring Assistant), and a Fundamental Administration DVD. Cost U$ 1592,00 (R$ 4968,00*).
Bayley™–III: Combination Cognitive/Language/Motor Record Form Pkg of 25=U$169.0/unit (4 pkg;U$ 676,00) Total cost R$2110,00*
Bayley™–III Motor Scale: Motor Record Form Pkg of 25=U$107.00/unit (4 pkg; U$428,00). Total cost R$1336,00*
Bayley™–III: Social-Emotional and Adaptive Behaviour Questionnaire Pkg of 25=U$144.00/unit (4 pkg;U$576,00).Total cost R$1798,00*
Bayley™ –III: Caregiver Report Form Pkg of 25= U$107.00 /unit (4 pkg U$428,00).Total cost R$ 1335,00* Bayley™–III: Stimulus Book cost=U$406.00/ Unit .R$ 1267,00*
Bayley™–III: Picture Book cost = U$34.00/Unit.R$107,00* The total cost of BayleyScales instruments is R$ 12921,00* (* exchange calculated value in 05/25/2015)
TOTAL Equipment for AIMS e Bayley III scales (year one): R$14589,00
AIMS Examiner Qualifications: Must be administered by professionals in child healthcare who have knowledge of normal infant motor development and experience with administering the instrument. Total estimated cost for applications by physiotherapists is R$ 16.800,00, both arms, IG and CG.
BAYLEY Examiner Qualifications: Must be administered by physiologists with formal training and experience with these neurodevelopment scales.

C.Biographical Information

1. March of Dimes, PMNCH, Save the Children, WHO. Born too Soon: The global action report on preterm birth. World Health Organization. Geneva, 2012.

2. Guinsburg R, de Almeida MF, de Castro JS, Silveira RC, Caldas JP, Fiori HH, ET AL. Death or survival with major morbidity in VLBW infants born at Brazilian neonatal research network centers.J Matern Fetal Neonatal Med. 2015; 2:1-5.
3. Spittle A, Orion J, Anderson P, Boyd R, Doyle LW. Early developmental intervention programmes post-hospital discharge to prevent motor and cognitive impairments in preterm infants. Cochrane Database of Systematic Reviews 2012; issue 12 Art n :CD005495.
4. Melnyk BM, Alpert-Gillis L, Feinstein NF, Fairbanks E, Czarniak-Schultz N, Hust D, et al. Improving cognitive development of low-birth-weight premature infants with the COPE program: a pilot study of the benefit of early NICU intervention with mothers. Research in Nursing and Health 2001; 24(5):373–89.
5. Field T. Preterm infant massage therapy studies: an American approach. Semin Neonatol 2002; 7: 487–494.
6. Bond C. Positive touch and massage in the neonatal unit: a British approach. Semin Neonatol 2002; 7: 477–486.
7. Litmanovitz I, Dolfin T, Friedland O, Arnon S, Regev R, Shainkin-Kestenbaum R et al. Early physical activity intervention prevents decrease of bone strength in very low birth weight infants. Pediatrics 2003; 112: 15–19.
8. Mendes EW, Procianoy RS. Massage therapy reduces hospital stay and occurrence of late-onset sepsis in very preterm neonates .J Perinatol 2008; 28:815-830.

9. Terra L, Hauck S, Schestatsky S, Fillipon AP, Sanchez P, Hirakata V, Ceitlin LH. Confirmatory factor analysis of the Parental Bonding Instrument in a Brazilian female population. Aust N Z J Psychiatry. 2009 ;43(4):348-54.
10. Procianoy RS, Mendes EW, Silveira RC. Massage therapy improves neurodevelopment outcome at two years corrected age for very low birth weight infants. Early Hum Dev. 2010; 86 (1):7-11.
11. Valentini NC , Saccani R. Escala Motora Infantil de Alberta: validação para uma população gaúcha . Rev Paul Pediatr 2011; 29 (2):231-8.
12. Bayley N. Manual for the Bayley Scales of Infant and Toddler Development. 3rd ed. San Antonio, TX: Psychological Corporation; 2006
13. Spittle AJ, Lee KJ, Spencer-Smith M, Lorefice LE, Anderson PJ, Doyle LW. Accuracy of Two Motor Assessments during the First Year of Life in Preterm Infants for Predicting Motor Outcome at Preschool Age. PLoS One. 2015;10(5):e0125854.
14. Marlow N. Neurocognitive outcome after very preterm birth. Arch Dis Child Fetal Neonatal Ed 2004;89:F224–F228.
15. Follow-up care of high-risk infants. Pediatrics 2004; 114:1377– 1397.
16. Vohr BR, Wright LL, Poole WK, McDonald SA. Neurodevelopmental outcomes of extremely low birth weight infants.
17. Anderson P, Doyle LW; Victorian Infant Collaborative Study Group. Neurobehavioral outcomes of school-age children born extremely low birth weight or very preterm in the 1990s. JAMA 2003;289: 3264–3272.
18. Morgan C, Novak I, Badawi N. Enriched environments and motor outcomes in cerebral palsy: systematic review and meta-analysis.Pediatrics 2013;132 (3):e735-46.
19. Morgan C, Novak I, Dale RC, Badawi N. Optimising motor learning in infants at high risk of cerebral palsy: a pilot study.BMC Pediatr. 2015 Apr 1;15:30. doi: 10.1186/s12887-015-0347-2.
20. Morgan C, Novak I, Dale RC, Guzzetta A, Badawi N.GAME (Goals - Activity - Motor Enrichment): protocol of a single blind randomised controlled trial of motor training, parent education and environmental enrichment for infants at high risk of cerebral palsy.BMC Neurol. 2014 Oct 7;14:203. doi: 10.1186/s12883-014-0203-2.
21. Roberts MY, Kaiser AP, Wolfe CE, Bryant JD, Spidalieri AM.Effects of the teach-model-coach-review instructional approach on caregiver use of language support strategies and children's expressive language skills.J Speech Lang Hear Res. 2014 ; 57(5):1851-69.

Ministério da Saúde

Ministério da Ciência, Tecnologia e Inovação

ANEXO I

CHAMADA MCTI/CNPq/MS/SCTIE/Decit/Fundação Bill e Melinda Gates N º 47/2014

Grandes Desafios/Grand Challenges Brasil
Formulário de Propostas e Instruções

Data de Submissão: 30/08/2015

Informações da Proposta

A. Organização

Nome da Organização: Hospital de Clinicas de Porto Alegre

Funcionário autorizado a submeter propostas e aceitar recursos em nome da organização:

Título	*Prof Dr* *Rita de Cassia*	Sobrenome	Silveira MD
		Telefone	5133315726/5198667912
Endereço	*Rua Silva Jardim 1155 apto 701.Porto Alegre-RS*	*Fax*	51 33289270
E-mail	drarita.c.s@gmail.com		
Website	rcsilveira@hcpa.edu.br		

B. Projeto

Nome do Projeto: **Programa de estimulação precoce para crianças prematuras e seus pais: estabelecendo o impacto no neurodesenvolvimento aos 18 meses de idade corrigida**

Investigador principal/ Coordenador do projeto:

		Sobrenome	*Silveira*
Título	Prof Dra Rita de Cassia	*Telefone*	5133315726/5198667912
			51 33289270
		Fax	
Endereço	*Rua Silva Jardim 1155 apto 701.Porto Alegre-RS*		
E-mail	drarita.c.s@gmail.com		
Website	rcsilveira@hcpa.edu.br		

Valor solicitado (Reais): R$ 486548,00 **Duração do Projeto (meses):** 24 meses

Estimativa do custo total do projeto R$ 486548,00

(Reais): ____________________

Receita total da organização para o último ano fiscal auditado (Reais): R$ ____________________

Índice:

I. Objetivo Principal ____ 4
II. Sumário Executivo ____ 4
III. Contexto ____ 7
IV. Estrutura do Projeto ____ 9
V. Capacidade Organizacional e de Gestão ____ 11
VI. Citações ____ 13
VII. Apêndices ____ 13
  A. Planilha de Orçamento ____ 13
  B. Detalhamento Orçamentário ____ 14
  C. Informações Biográficas ____ 16

<u>I Objetivo Principal</u>

Estabelecer um programa de intervenção precoce para crianças nascidas prematuras de forma a possibilitar a execução dessa intervenção de forma continuada e sistemática pelas famílias. Desenvolver uma forma de quantificar a resposta a estimulação da cognição e da motricidade em conjunto é um objetivo adicional dessa pesquisa.

<u>II Sumário Executivo</u>

A prematuridade e suas conseqüências causam grande impacto na sociedade e nos indicadores de saúde de uma população. Dados da Organização Mundial da Saúde indicam que o Brasil está na décima posição entre os países onde mais nascem prematuros e na décima sexta posição em mortalidade neonatal, com prematuridade contribuindo para 45% das mortes entre os recém-nascidos. No ano de 2012 houve cerca de três milhões de nascimentos ao ano no Brasil, trezentos e cinqüenta mil com menos de 37 semanas, sendo que o índice de nascimentos prematuros nos últimos três anos aumentou, incluindo prematuros de muito baixo peso (idades gestacional inferior a 32 semanas e peso de nascimento abaixo de 1500 gramas). Nos centros da Rede Brasileira de Pesquisas Neonatais, prematuros com peso de nascimento inferior a 1 500 gramas apresentaram mais de 50% de mortalidade ou sobrevida com morbidades maiores[1,2].Dentre os sobreviventes há necessidade de acompanhamento ambulatorial após a alta.O seguimento após a alta da Neonatologia é uma extensão dos cuidados perinatais e deve oferecer condições de monitorizar o crescimento, desenvolvimento e as morbidades comuns, com equipe multidisciplinar capaz de avaliar integralmente a criança e o contexto à mesma está inserida.

Intervenção precoce para o prematuro de risco deve apresentar-se com uma interação dinâmica entre o recém-nascido, família e o ambiente ao redor[3]. É possível que um processo de cuidado modificado por parte das famílias seja benéfico para o neurodesenvolvimento do prematuro, tanto no aspecto cognitivo quanto motor.

<u>Justificativa</u>

As crianças nascidas prematuras apresentam risco elevado de atraso no neurodesenvolvimento cognitivo e motor. Diversos programas de intervenção foram previamente descritos na tentativa de melhorar os desfechos neurológicos dessas crianças, mas de acordo com revisão da Cochrane há grande heterogeneidade entre os estudos e variedade de intervenções incluindo diferentes idades gestacionais [3] .

Programas de intervenção precoce que focam no desenvolvimento desde a UTI Neonatal e após a alta do hospital para a comunidade podem ter um impacto mais significativo nas morbidades em longo prazo as quais esses prematuros estão mais suscetíveis. Intervenções com objetivo de melhorar a relação parental, focando na sensibilidade dos pais para perceberem o momento mais adequado para um determinado estímulo para a criança, adequando esse estímulo à necessidade das mesmas, possivelmente influenciarão na melhor cognição e desenvolvimento social[3,4].

Apesar dessas evidencias, o envolvimento parental na qualidade e continuidade desses estímulos é pouco avaliado e validado, especialmente em populações de nível social e econômico desfavorecido.

<u>Executivo da estruturação do projeto</u>

Devido a importância de atuar na janela de maior plasticidade neuronal, ou seja, primeiros 12 meses de idade corrigida, as crianças nascidas em nossa instituição serão incluídas com 48 horas após nascimento na internação neonatal (primeira intervenção e randomização) serão acompanhadas e na fase pré alta ainda na neonatologia (segunda intervenção) será a avaliado vínculo parental.Após a alta hospitalar serão encaminhadas ao ambulatório de seguimento e conforme randomização previa em dois braços:

1.Programa padrão de avaliação motora e cognitiva e intervenção de acordo com as demandas diagnosticadas

2.Programa de intervenção antecipada em estímulos orientados aos familiares, independente da avaliação padrão estipulada.

Todas as facetas do neurodesenvolvimento infantil serão devidamente avaliadas e antecipadamente estimuladas pelos pais: motor, linguagem e cognição de forma indissociável e interativa, o que é inovador.

Randomização: os neonatos prematuros serão sequencialmente randomizados ao completar 48 horas de vida.

O grupo 1 (grupo convencional-GC) receberá o cuidado padrão, de acordo com a rotina assistencial da UTI Neonatal.

O grupo 2 (Grupo Intervenção-GI) receberá estimulação tátil e cinestésica realizada pelas mães até a alta hospitalar. O planejamento da intervenção realizada exclusivamente pela mãe foi baseado em estudos que aplicaram estimulação tátil e exercícios passivos em prematuros[5-7].
Todos os grupos recebem o cuidado pele a pele (Kangaroo Care) de acordo com a rotina assistencial.
Em estudo prévio, realizamos a mesma intervenção na Neonatologia , como segue[8]:
Orientação para as mães realizarem uma seqüência de 15 minutos de estímulos, quatro vezes ao dia com intervalo de seis horas. A estimulação tátil será realizada nas superfícies cutâneas e área muscular das regiões da face correspondente ao temporal, frontal periorbital, nasal e perilabial e no lado externo dos membros superiores e inferiores. As mães usarão dois a três dedos agrupados que são passados suavemente com pressão moderada, até três vezes em uma direção e três vezes na direção oposta.A estimulação cinestésica consiste em exercícios passivos (flexão e extensão) de membros superiores e inferiores, um membro por vez e até três vezes em cada articulação (pulso, cotovelo, tornozelo e joelho); uma das mãos fica apoiada no membro que está sendo estimulado e a outro realiza o movimento de flexão e extensão[8].
Para garantir uma aplicação segura da intervenção, as mães randomizadas para grupo 2, serão instruídas a observar os sinais vitais, a tolerância dos recém-nascidos e a evitar estímulos excessivos.
Um pesquisador terá reuniões regulares com as mães incluídas no Grupo Intervenção a cada 48 horas para assegurar que elas estejam fazendo a intervenção como instruídas, assim como avaliação da interaçao[9] .
Em publicação prévia, evidenciamos que esse protocolo de massagem terapia realizado pelas mães combinado com o contato pele a pele padrão melhorou o neurodesenvolvimento aos dois anos de idade corrigida[10].
Na alta hospitalar, será procedido uma avaliação do nível de stress parental e vínculos pais –bebê em todos os recém-nascidos elegíveis para o estudo e sobreviventes, sendo o segundo momento de intervenção.Psicóloga cegada para qual grupo a mãe pertence fará a avaliação usando o PARENTAL BONDING INSTRUMENT (PBI)[9].
As seguintes variáveis sócio –demográficas e neonatais serão coletadas em ambos os grupos para estabelecer comparações: idade materna e paterna, nível de escolaridade materna que será obtido mediante aplicação de questionário diretamente com o cuidador principal nas visitas do seguimento ambulatorial;peso de nascimento e idade gestacional; peso no momento da alta ou com 40 semanas de idade corrigida (o que acontecer primeiro); e aos 12,18,24 meses (idade corrigida);adequação do peso para idade gestacional ao nascimento, na alta ou nas 40 semanas de IC (o que acontecer primeiro); numero de crianças com perímetro cefálico abaixo do percentil 10 ao nascimento, na alta ou nas 40 semanas de IC (o que acontecer primeiro); velocidade de crescimento somático do nascimento até a alta ou nas 40 semanas de IC (o que acontecer primeiro); presença de sepse precoce e tardia, enterocolite necrosante, sindrome do desconforto respiratório, hemorragia cerebral (graus II, III e IV),Leucomalácia Periventricular cística e não cística, retinopatia da prematuridade, displasia broncopulmonar (leve, moderada ou grave). A idade gestacional será determinada pelos dados maternos (DUM), confirmada por ultrassonografia obstétrica precoce ou exame fisico neonatal. No seguimento, serão avaliados número de crianças com peso, comprimento e perímetro cefálico menos que dois desvio padrão da mediana de referencia (escore Z) nas curvas da Organização Mundial da Saúde aos 12, 18 , 24 meses de idade corrigida.
Seguimento ambulatorial, intervenção e visitas domiciliares
Todos os prematuros com peso de nascimento inferior a 1500 gramas são rotineiramente encaminhados ao ambulatório de seguimento para consultas mensais nos primeiros 6 meses de idade corrigida, bimensais de 7 meses a 12 meses de idade corrigida, e depois trimestralmente até completar 24 meses de idade corrigida. Conforme randomização prévia, o grupo 1 terá: programa padrão de avaliação motora e cognitiva e intervenção de acordo com as demandas diagnosticadas e para o grupo 2 (GI), orientações serão administradas aos pais e/ou cuidadores de forma sistematizada em todas as consultas de seguimento; mensal no primeiro semestre e bimensal no segundo semestre até 12 meses de idade corrigida, a partir de então, mais duas orientações trimestrais.
Programa de orientação sistematizada será de acordo com marcos do desenvolvimento, antecipando em um mês a etapa evolutiva de aquisição motora e/ou cognitiva esperada para a idade corrigida.No primeiro semestre de idade corrigida, a mãe, pai e /ou cuidador correspondente receberá orientações simples para estimular motricidade ampla, fina e cognição.
Até três meses de idade corrigida: estimulação na posição deitada na cama.Estimulação motora ampla e fina: Cruzamento de braços e movimentos de descontração (brincar no banho por 5 a 10 minutos, batendo mãos e pés na água, bicho de borracha). Movimentos de ginástica (flexão e extensão passiva

dos membros superiores e inferiores) com finalidade da criança tomar consciência do corpo.Estimulação cognitiva: Ficar bem perto e falar devagar, cantar baixo. Mobile para olhar para cima, luvas para colocar nas mãos da mãe de cor preto e branca para brincar e olhar virando a cabeça 180◦C. Entre 3 e 6 meses de idade corrigida:Estimulação motora ampla e fina: deitar a criança de costas para uma bola grande plástica, segurando-lhe as coxas. Inclinar leve e lentamente a bola pra frente e para trás, para um lado e outro, com a finalidade de preparar para a posição sentada e obter equilibro. Deitada de costas com um móbile na altura da barriga, braços esticados em cruz e mãe segura as mãos da criança, mostrando a ela que deverá tentar pegar com os pés, aprendendo a pedalar. Ensinar a tocar objetos com diferentes texturas (macio, duro, áspero, rugoso) descrevendo a característica do toque (estimula junto cognição). Estimulação cognitiva: ouvir músicas, cantar e leitura simples de palavras e frases curtas. Após o banho, e enquanto seca com a toalha partes do corpo, a mãe fala: enxugue o pé, a mão, o braço e assim por diante, falando pausadamente com a criança. No espelho, mostrar e nominar: olhos, boca, nariz da criança e da mãe, para a mesma adquirir consciência de sua individualidade.

Dos seis aos 12 meses de idade corrigida:Estimulação motora ampla e fina: a ginástica pretende tonificar a musculatura a fim de preparar primeiro para a posição sentada e depois em pé sem apoio, até caminhar independente. Usando uma bola grande de plástico (a bola não deve estar muito cheia) a mãe será orientada a segurar a criança contra si, uma mão segurando os joelhos da mesma e a outra o peito. Inclinar o peito da criança em direção ã bola até que ela se apóie nas mãos e faça um leve esforço para se erguer. Na seqüência dos meses, rodar a bola , fazendo com que a criança solte o seu ponto de apoio com uma mão , depois a outra e por fim as duas.Importante orientar a mãe a liberar o quadril da criança progressivamente.Usar o chão com uma espécie de tapete com material EVA para a criança ter espaço de deslocamento.Para estimular motricidade fina, orienta-se a oferecer revistas para a criança rasgar, demonstrando como fazê-lo, calmamente. Uma vez rasgada em vários pedaços, ensinar a fazer uma bola de papel com os pedaços e jogar bola com a criança. O tempo dos exercícios não deve ser superior a 15 minutos e devem parecer brincadeiras, na freqüência obrigatória de três vezes/semana.

Estimulação cognitiva: bolas de cor preta e branca; e na seqüência bolas coloridas, sempre no mesmo lugar para a criança achar mais fácil. Conversar repetidamente reforçando cada conquista da criança. A partir de 8 meses de IC, mostrar e nominar as partes do corpo: cabeça, barriga, mão, pé, boca, nariz, olhos.. Ler livros e mostrar as figuras com os animais e objetivos, repetindo o nome de cada figura bem pausado (silabicamente) e com a boca bem aberta.

O tempo dessas atividades não deve ser superior a 15 minutos e devem parecer brincadeiras, na freqüência obrigatória de três vezes/semana (intercalando com a estimulação motora ampla e fina).

Após 12 meses de idade corrigida:Estimulação cognitiva, motor fino e ampla: nessa fase todo processo é integrado e continuo, explorando as habilidades individuais de cada criança: para cantar, falar, contar estórias...Deve envolver a alimentação com colher, nomes aos alimentos, ensinando a criança a falar e reforçando cada conquista. Alimentos que seja possível a criança levar à boca de forma independente. Ensinar a fazer desenhos em folhas com giz de cera coloridos, falando o tempo todo com a criança. Os movimentos globais serão jogos que precisam da adesão total da criança (briquedos coloridos de encaixe, bola, boneco de pano). Na motricidade ampla, é fundamental usar dois bastões de madeira e tentar caminhar com esse apoio e mais tarde subir escadas.

Nessa fase, a mãe ou cuidador principal deve ser encorajada a manter a estimulação diária, por 10 a 15 minutos.

Devem ser realizadas duas orientações trimestrais para estímulos cognitivos, motor fino e amplo, totalizando as 10 sessões domiciliares de orientações e supervisão.

Estimam-se de acordo com o numero de orientações realizadas em nível ambulatorial, o mesmo numero de visitas domiciliares (10 visitas) com questionário e checagem das informações recebidas, com a finalidade de avaliar a compreensão e efetivo seguimento por parte das famílias dessas orientações.

Análise do impacto da intervenção (avaliação final) no grupo intervenção e controle (garantia de cegamento dos avaliadores).

As avaliações finais do neurodesenvolvimento motor e cognitivo serão realizadas mediante aplicação da escala AIMS e Bayley III aos 12 a18 meses de idade corrigida em ambos os braços.

Escala AIMS (Alberta Motor Infant Scale): Fisioterapeuta cegada para o grupo ao qual a criança foi alocada realizará aos 12 a 18 meses a aplicação da escala motora infantil de Alberta em todos os pacientes elegíveis para a pesquisa. A avaliação será realizada na presença dos pais ou cuidadores em uma sala com superfície segura para a criança movimentar-se durante a avaliação. O examinador irá interagir com a criança para encorajar a resposta, mas a facilitação física de movimento deve ser evitada. Durante a avaliação, são pontuados os comportamentos mais ou menos maduros dentro do repertório motor da

criança em cada postura (supino, prono, sentado e em pé). Este repertório é denominado de "janela motora". Todos os itens observados dentro da janela motora e os itens anteriores à janela motora são pontuados. Ao término da avaliação, a criança receberá uma pontuação baseada na soma dos itens pontuados em cada postura, denominada escore bruto. Este escore será observado em um gráfico de padronização a fim de encontrar o percentil de desenvolvimento do bebê, conforme a idade cronológica ou corrigida. Os percentis padrões do instrumento são: 5%, 10%, 25%, 50%, 75%. De acordo com este percentil o desenvolvimento do bebê pode ser classificado em três categorias: normal ou típico (percentil>25%), suspeito (percentil>5% e ≤25%), anormal ou atípico (percentil≤5%)[11].
BSDI-III: *Bayley Scales of Infant and Toddler Development* na Terceira versão é uma escala que considera os três componentes: linguagem . cognição e motricidade. Usada para avaliar o neurodesenvolvimento aos 12-18 meses de idade, em todos as crianças incluídas no estudo, durante a consulta de rotina no programa de seguimento, por psicóloga sem conhecimento para o qual grupo foi alocado o sujeito. Os escores gerados por cada área serão considerados neurodesenvolvimento: normal quando superior a 89; atraso leve de 80-89; limítrofe se 70 a 79; e extremamente atraso quando inferior a 69. Os casos de cegueira e surdez irreversível, serão considerados atraso extremo do neurodesenvolvimento, de acordo com recomendação do manual[12]. A aplicação da escala Bayley permite exame global do neurodesenvolvimento da criança.
O uso de dois testes motores e em mais de um momento ; AIMS e Bayley, é justificado porque falsos positivos são comuns , havendo beneficio também no seguimento continuado e em mais de um momento[13].
As taxas de prevalência de aleitamento materno exclusivo e aleitamento misto com 6 meses de idade corrigida serão avaliadas e anotadas, como uma medida do vínculo materno, em ambos os braços.
Avaliação da interação pais-bebe será realizada pré e pós-intervenção para todos os pacientes, mediante a aplicação do *Parental Bonding Instrument* [9].
Parental Bonding Instrument (PBI):consiste em questionário com 25 itens auto-avaliados pelos pais ë validada no Brasil..O instrumento mede dois constructos: o primeiro é a afeição, que é mais consistente e claramente bipolar (afeto , calor, disponibilidade , cuidado, sensibilidade em relação frieza e rejeição) ; e o segundo é o controle ou proteção (controle, contra intrusão encorajamento de autonomia) .
Com uma ano de idade cronológica será realizada uma avaliação global do neurodesenvolvimento para todas as crianças incluídas no estudo.
Todo o processo dessa pesquisa conta com a participação de equipe multidisciplinar.
Se este programa inovador, de intervenção precoce e global continuada e realizada pelas mães, evidenciar bons resultados no neurodesenvolvimento dos prematuros, planejamos ofertar ara toda a população , uma vez que é uma proposta simples de ser captada pelas equipes de saúde.

III Contexto

Em países de condições econômicas e diversidade social menos favorecida é importante adequar a alocação do recurso. As crianças prematuras necessitam acompanhamento com um perfil de profissionais capacitados para proporcionar uma extensão dos cuidados perinatais, oferecendo condições de adequado acompanhamento quanto ao crescimento, neurodesenvolvimento e morbidades comuns a esta população de maior risco. As necessidades são múltiplas, razão pela qual, o atendimento deve ser multidisciplinar e deve ser estabelecido um ponto de corte de idade gestacional e peso de nascimento onde a necessidade é maior e mais significativa: para essa pesquisa o ponto crítico escolhido com base em diversos desfechos da literatura foi a faixa de idade gestacional inferior a 32 semanas ou aqueles prematuros nascidos com muito baixo peso, peso de nascimento inferior a 1 500 gramas, independente da idade gestacional[14-17].

O ambulatório de seguimento de prematuros existente de forma sistematizada em nossa Instituição acompanha em consultas periódicas com avaliação do desenvolvimento e do crescimento e encaminha conforme a necessidade e identificação de déficits para realização de estímulos os pacientes egressos da UTI Neonatal com peso de nascimento inferior a 1500 gramas ou idade gestacional inferior a 32 semanas. A abordagem multiprofissional com intervenções precoces que possam resultar em melhor desempenho e qualidade de vida no futuro para estas crianças deve ser estruturada e coordenada pelo neonatologista e/ou pediatra que tenha a visão do todo do programa.

Há múltiplas situações as quais os prematuros são vulneráveis, os problemas e lesões neurológicas, déficit de crescimento, problemas psiquiátricos, déficit visual e auditivo, motricidade ampla e fina, linguagem . No contexto da pesquisa encontra-se a antecipação dessa situação com estimulação de forma independente ao atraso detectado nas avaliações, é uma intervenção sistemática precoce e adicional aquela já instituída na rotina assistencial, conforme publicação prévia[10].

A equipe será composta pelos seguintes profissionais e sua funções abaixo expostos no quadro :

Equipe do programa de seguimento

Membro da equipe	Papel na equipe de seguimento
Pediatra/neonatologista	Núcleo da equipe, coordenação.<br>Avalia crescimento e triagem do desenvolvimento<br>Responsável pelo manejo de intercorrencias clínicas em geral
Psicóloga infantil	Avaliação formal do neurodesenvolvimento com escalas diagnósticas<br>Triagem dos problemas comportamentais, vínculos e manejo dessas situações.<br>Intervençoes de apoio e suporte terapêutico das morbidades psiquiátricas frequentes
Neurologista pediátrico	Manejo em longo prazo das neuropatologias, tais como convulsões, paralisia cerebral, distúrbios de deglutição
Oftalmologista e retinólogo	Follow-up da retinopatia da prematuridade e tratamento<br>Avaliação da acuidade visual e triagens tais como: estrabismo, nistagmo, erros de refração.<br>Prevençao da cegueira
Otorrinolaringologista	Manejo da perda auditiva
Nutricionista clínica	Conselhos aleitamento materno e alimentação complementar adequada<br>Manejo das crianças com falha de crescimento<br>Manejo das situações que requerem dieta especial; ex galactosemia.
Fonoaudiologia	Avaliar potencial auditivo, prevenção da perda auditiva, coordenação da deglutição e auxilio nas dificuldades de linguagem e fonação.
Enfermagem	Orientaçao higiene,aspectos gerais da puericultura, checagem das medicações e imunizações.
Equipe para visitas domiciliares	Pediatra, neuropsicologo, fonoaudiólogo, fisioterapeuta, nutricionista, enfermagem e terapeuta ocupacional

A hipótese principal da pesquisa é que uma intervenção com estimulação precoce continuada realizada pelos pais e focada nos aspectos globais do neurodesenvolvimento infantil é mais efetiva que apenas a intervenção tradicionalmente proposta. Muitos estudos de intervenção focam mais na avaliação da efetividade do domínio motor do que na cognição, porque é mais factível a mensuração do efeito[18]. Literatura recente sobre neuroplasticidade sugere que intervenções intensivas, como tarefa-específica devem começar o mais cedo possível e em um ambiente enriquecido, durante o período crítico de desenvolvimento neural. Intervenções motoras ativas são eficazes em algumas populações, no entanto os efeitos de intervenções motoras ativas sobre os resultados na motricidade das crianças com paralisia cerebral têm sido pesquisado apenas em um estudo piloto[19]. Esse estudo usou o protocolo GAME (Goals - Activity - Motor Enrichment)[20]. A cognição é pobremente avaliada nesses programas de intervenção. O que há descrito é o efeito da abordagem instrucional Teach- Model- Coach- como estratégia de apoio linguístico aos cuidadores, empregando-se com os filhos uma linguagem expressiva com resultados promissores, mas preliminares[21.]
Desenvolver uma forma de quantificar a resposta à estimulação da cognição e da motricidade em conjunto é um objetivo adicional dessa pesquisa.
Ë conhecido que programas de intervenção precoce para crianças prematuras apresentam uma influência positiva nos desfechos motores e cognitivos. Há evidencias de que interações pais-filhos de alta qualidade exercem influencia positiva no neurodesevolvimento social e cognitivo da criança[9,14,17].
Como o seguimento ambulatorial é uma extensão dos cuidados perinatais, esse grupo de profissionais da saúde, acompanhará a criança e sua família desde durante internação neonatal , próximo a alta hospitalar será checado o vinculo , a equipe visa o preparo para o seguimento ambulatorial, trabalhando a redução do stress parental, vínculos pais-bebes e nas etapas evolutivas do seguimento, estímulos específicos para aspectos motores, cognitivos e de linguagem.
Fisioterapeuta cegada para o grupo ao qual a criança foi alocada realizará aos 12 a 18 meses a aplicação da escala motora infantil de Alberta em todos os pacientes elegíveis para a pesquisa.
Psicóloga do grupo de pesquisa cegada para o grupo ao qual a criança foi alocada aplicará a escala Bayley versão III de avaliação do neurodesenvolvimento em todos os pacientes elegíveis para a pesquisa.

Ambos os grupos receberão os estímulos padrão da equipe multiprofissional desde a internação neonatal (Canguru) e do seguimento ambulatorial. O grupo II receberá estímulos adicionais especificos ofertados pela mãe desde as 48 horas após nascimento e visitas domiciliares em numero de 10, assim como orientações para estímulos domiciliares que serão averiguados nessas visitas domiciliares da equipe multiprofissional.
Avaliação da interação pais-bebe será realizada pré e pós-intervenção para todos os pacientes[9].
Dr Renato S Procianoy MD PhD e Dra. Rita C Silveira MD PhD são os investigadores principais, com a colaboração do T. Michael O'Shea, MD, MPH **.** Esses pesquisadores têm muitos estudos anteriores publicados, onde a relação entre as morbidades dos pré-termos e desfechos do neurodesenvolvimento foram avaliados. Dr O'Shea tem extensa experiência com estudos conduzidos pelo NICHD e atualmente é um dos coordenadores do estudo ELGAN (Extremely Low Gestational Age Newborns).

IV Estrutura do Projeto

Tabela da estrutura do projeto

	**Resultados**	**Período de atividade**
**Objetivo 1:** Título. Criação de programa de intervenção precoce para crianças nascidas prematuras de forma continuada e sistemática realizado pelas suas famílias.	Melhora da interação dos pais com bebe, precocemente, desde a internação neonatal, com a mãe envolvida no cuidado e provendo estimulação . <br>Redução do stress e ansiedade parental <br>Impacto no melhor desempenho global no neurodesenvolvimento do prematuro avaliado aos 18 meses de idade corrigida	TODO O PERIODO DO ESTUDO. Iniciará em um mês após aprovação do recurso com organização e aplicação de protocolos para preparo da equipe: ensinar as massagens e estimulação tátil e cinestésica para as mães.Preparar a equipe na organização para a alta dos prematuros; reuniões da equipe multiprofissional com as famílias Toda equipe mutiprofissional deverá ser treinada quanto ao tipo de orientação a ser ministrada às famílias. Participantes serão logitudinalmente captados e randomizados sequencialmente a partir do momento que completarem 48 de vida, até um número total de 84 sujeitos. Antes da randomização serão excluídos os pacientes com malformações congênitas maiores, síndromes genéticas e aqueles que os pais recusarem participar do estudo . <br>Nas avaliações, ao final, profissionais com capacitação para testagens realizarão as avaliações do desenvolvimento e divulgarão os resultados .
**Objetivo 2:** <br>Promover orientação e educação dos cuidadores quanto às necessidades da criança em cada etapa evolutiva das mesmas	Baixas taxas de falha no seguimento (busca ativa) <br>Redução do stress parental (medido previamente)	Após o inicio do financiamento, serão randomizados para um grupo receber cuidado padrão e outro cuidado padrão mais intervenção proposta. Na internação a intervenção

	Melhora do vinculo pais bebes (medido pela equipe multiprofissional)<br><br>Visitas domiciliares para comprovação da realização dos estímulos	promoverá um momento de cuidado e interação adicional por meio da massagem terapia realizada pelas mães. No seguimento consistirá em orientações e medidas educativas aos pais que promoverão estimulação precoce em casa de forma continuada (diária, 10, 15 minutos/dia) além da orientação padrão da equipe de seguimento.
**Objetivo 3:**<br><br>**Avaliar o impacto das medidas instituídas no melhor desempenho no neurodesenvolvimento**	Vínculos mais fortalecidos que no inicio do programa, medido pelo PBI resultando em maior escore de atenção, cuidado e proteção.<br><br>Aquisições motoras na faixa de idade corrigida ou cronológica conforme escala Alberta.<br><br>Linguagem com padrão bem estabelecido para idade. Comunicabilidade e sociabilidade presentes e de forma mais evidente que o grupo cuidado padrão, resultado em escala Bayley com escores de cognição, linguagem e motricidade próximos ao normal em número maior que o esperado, baseado em dados de pesquisas prévias.<br><br>Medidas estatísticas das diferenças entre os grupos (intervenção e convencional abordagens).<br><br>.	Iniciará no momento da alta hospitalar (PBI) e no seguimento, após as 10 sessões domiciliares de orientações, com 18 meses de idade corrigida e concluirá após realização das avaliações do desenvolvimento; que se estenderá até 12- 18 meses de idade corrigida com a avaliação motora (AIMS e BAYLEY), cognitiva (BAYLEY) e Linguagem (BAYLEY) ainda, de vínculos parentais (PBI).<br><br>Uma avaliação global do neurodesenvolvimento será realizada com 1 ano de idade cronológica, para ambos os grupos.

Atividades:

Ao completar 48 horas de vida o prematuro de muito baixo peso (peso de nascimento inferior a 1500 gramas e idade gestacional inferior a 32 semanas) nascido no HCPA, que não apresenta critérios de exclusão, será elegível para o estudo. Assim, a primeira atividade será obter consentimento informado escrito e assinado pelos pais, para possibilitar a randomização.

A randomização será realizada por pesquisador que não será responsável pela intervenção, nem a medidas de desfechos. É o mesmo pesquisador que anotará dados clínicos da internação neonatal, da alta e encaminhará ao seguimento clínico. O alocamento dos sujeitos será mantido até completar o cálculo amostral, baseado no número de altas hospitalares desses prematuros que sobrevivem ao termo equivalente ou momento da alta, sendo rotineiramente candidatos ao seguimento ambulatorial.

Um pesquisador treinado avaliará na alta hospitalar o nível de stress parental e vínculos pais –bebê em todos os recém-nascidos elegíveis para o estudo e sobreviventes. Profissional cegado para qual grupo a mãe pertence fará a avaliação usando o PARENTAL BONDING INSTRUMENT (PBI)[9].

Equipe mutidisciplinar prepara os elegíveis para a alta, avaliação de vínculos pela psicologia, promove reuniões com orientação padrão de alta para todos os pacientes; seguido de dois grupos de cuidados conforme a randomização prévia em dois braços:

1.Programa padrão de avaliação motora e cognitiva e intervenção de acordo com as demandas diagnosticadas

2.Programa de intervenção antecipada em estímulos orientados aos familiares, independente da avaliação padrão estipulada

Na etapa inicial vinculo parental será avaliado e trabalhado para estimular o maior apego em todos os momentos de avaliação.

Um número de três pediatras/neonatologistas estarão realizando consultas mensais no ambulatório de seguimento até seis meses de idade corrigida, de 2/2meses até 12 meses de idade corrigida e 3/3 meses até 18 meses de idade corrigida, de acordo com a rotina assistencial. Serão realizadas visitas domiciliares periódicas sistemáticas de acordo com o grupo randomizado, de toda a equipe multiprofissional. Os estímulos orientados para a família realizar serão checados nessa visitas.

A atividade de ensino e educação à família será o foco de todo o grupo de pesquisa, durante todo o estudo.

Nós ofereceremos condições de estabelecer estimulação precoce com protocolo de acordo com a idade corrigida, exercida pela família.

Estímulos visuais, auditivos, motor amplo e fino, socialização, definição e conhecimento de partes do corpo serão trabalhados conforme previamente descrito nesse projeto.

Resultados / marcos críticos

O maior resultado que será utilizado para medir quantitativamente é o progresso relacionado ao objetivo principal : criação de programa de intervenção precoce para crianças nascidas prematuras de forma a possibilitar a execução dessa intervenção de forma continuada e sistemática pelas famílias. E adicionalmente, desenvolver uma forma de quantificar a resposta à estimulação da cognição e da motricidade em conjunto e de forma sustentada.

Para uma avaliação crítica do resultado, foram considerados como desfechos de interesse os progressos na aquisição motora e cognição que foram adquiridos após a intervenção em qualquer momento, desde que mensuráveis a um escore 20% superior.

Cálculo do tamanho da amostra: realizado com base em diferentes estudos que avaliaram melhora na aquisição motora ampla, fina ou na cognição ou linguagem com diversas ferramentas para avaliação e intervenção. Todos esses estudos obtiveram escores mínimos 20% de ganho após a intervenção precoce. Para um nível de significância de 5% e um poder estatístico de 80%, o tamanho da amostra de 84 sujeitos já seria suficiente e necessário para detectar três pontos de diferença nos escores de desenvolvimento entre os grupos[10,19,20,21]. A alocação dos sujeitos elegíveis para o estudo será mantida até completar o tamanho da amostra calculado, baseado no número de prematuros que sobrevivem até o momento da alta hospitalar. Será adicionada a estimativa de 20% (n=16) para possíveis perdas ou óbitos no seguimento. Um total de 100 prematuros constitui a estimativa final da amostra.

Ao final do período de financiamento é esperado diferença significativa nos indicadores de desenvolvimento infantil no grupo com intervenção hospitalar associada a intervenção continua da família (braço 2 da randomização) comparado com grupo apenas com a intervenção padrão da equipe multiprofissional (braço 1), espera-se um aumento em 35% nos escores de desenvolvimento.

Avaliação dos resultados

Monitorar o progresso da pesquisa é fundamental para garantir que todo o processo de trabalho e atividades serão alcançadas de acordo com as metas previamente estabelecidas. A escolha do método adequado para gerar a sequência de randomização é importante para a aferição dos resultados, por exemplo, devendo ser continuadamente monitorada.Toda a equipe de pesquisa precisa oferecer oportunidade para a aprendizagem motora e " treinamento", sendo que para os fins desta pesquisa foram consideradas formação inclusive de adaptações ambientais para melhorar a avaliação e como forma de promover um ganho específico na motricidade, que será objetivamente avaliado pelas escalas motora Alberta e pela Bayley III.Resultado de impacto envolve melhorar a interação pais-bebê , educar os pais sobre como devem ajudar no desenvolvimento de habilidades de seu filho , e proporcionar oportunidades de

aprendizagem ativa da motricidade também (atividade motora auto- gerada) . Parental Bonding Instrument (PBI) fará a aferição deste resultado.

As ferramentas para avaliação dos resultados são conhecidas e bem descritas na literatura: BSDI III Bayley Infant and Todler Developmental Index version III), AIMS (Alberta Infant Motor Scale) , PARENTAL BONDING INSTRUMENT (PBI) .

Bayley e AIMS oferecem resultados quantitativos (escores) a partir da avaliação.

Análise estatística: variáveis quantitativas serão expressas em média e desvio padrão ou mediana e amplitude interquartil, de acordo com a assimetria ou não de cada variável. T Student ou Mann Whitney testes serão empregados para comparar os grupos.

As variáveis qualitativas serão expressas como freqüências absolutas e relativas. Testes de Pearson e Qui-quadrado poderão ser usados para determinar associações entre variáveis categóricas, com resíduos ajustados em caso de significância estatística.

Haverá garantia do cegamento dos participantes para a equipe envolvida nas avaliações.
O envolvimento parental será avaliado em população menos favorecida economicamente.

V Capacidade Organizacional e Capacidade de Gestão

A. Capacidade Organizacional e Instalações: A abordagem hospitalar ocorrerá na UTI Neonatal do Hospital de Clinicas de Porto Alegre; onde será realizada a alocação dos grupos e da população a ser acompanhada durante a internação hospitalar e no seguimento. A Unidade Obstétrica do Hospital de Clínicas de Porto Alegre tem 150 partos com idade gestacional ≤ 32 semanas por ano. E uma Unidade de Terapia Intensiva Neonatal nível III com 20 camas, com ventilação convencional e de alta freqüência, tratamento com óxido nítrico, ecografia craniana e cardíaca a beira do leito disponível a qualquer momento do dia. A equipe assistencial é constituída de três neonatologistas certificados de plantão todo o dia. Há também uma equipe disponível para assistência ao parto em qualquer hora do dia.
O seguimento será realizado no Ambulatório da própria instituição e as visitas domiciliares deverão obedecer horário previamente agendado com as famílias, sempre em grupos e coordenado pelos investigadores principais (RCS e RSP) .
B. Gestão e Plano de Pessoal para este Projeto:

1.Gestão e Planejamento de Recursos Humanos para este projeto
A gestão e planejamento dos Recursos Humanos serão gerenciados pelos pesquisadores principais.
Dr. Procianoy e Dra. Silveira irão observar e assegurar o cumprimento dos Procedimentos Operacionais Padrão para este estudo.
Recursos Humanos
Médicos neonatologistas (3) da equipe para as consultas de seguimento e coordenação da randomizaçao e da pesquisa;
Psicólogos (3), sendo 1 para as testagens e avaliações e 2 para orientar intervenções aos pais;
Nutricionistas (3), para avaliação do crescimento
Fisioterapeuta (3), sendo 2 para os estímulos motores e 1 para aplicação de testagens;
Enfermagem (2), para a intervenção na UTI Neonatal até a alta hospitalar
Enfermagem (2) : orientações no seguimento e nas visitas domiciliares;
Fonoaudióloga (3), estimulação motora oral, testagem de linguagem e estimulação da mesma;
Terapeuta ocupacional (1), para a intervenção.
Alunos de Iniciação cientifica (2), para coleta dos dados, montagem do banco de dados perinatais, neonatais e do seguimento.
A equipe multidisciplinar para realizar as visitas domiciliares, incluirá: pediatras ou neonatologistas, psicólogas, enfermeiros, fisoterapeuta, fonoaudióloga, nutricionistas e terapeuta ocupacional .
<u>Capacidade dos Investigadores e da Instituição</u>
Investigadores Principais:
Rita C Silveira MD PhD: Professora Associada do Departamento de Pediatria e do Programa de Pós Graduação em Saúde da Criança e do Adolescente em nível de Mestrado e Doutorado da Faculdade de Medicina da Universidade Federal do Rio Grande do Sul. Pequisadora Cnpq, 2. Coordenadora do Ambulatório de Prematuros do Hospital de Clinicas de Porto Alegre, com experiência no seguimento clinico dos prematuros há mais de dez anos. Atual Chefe do Serviço de neonatologia do HCPA. Será responsável

pela coordenação e gestão dos dados dos pacientes, acompanhamento clinico, fiscalização do cegamento das intervenções no seguimento, coordenação da equipe multiprofissional no seguimento durante as consultas na instituição e nas visitas domiciliares . Com publicações em pesquisa clinica em Neonatologia explorando o mecanismo fisiopatológico das doenças e os diversos desfechos que nevolvem o seguimento de prematuros, com foco no crescimento de desenvolvimento.

Renato S Procianoy MD PhD: Professor Titular do Departamento de Pediatria e do Programa de Pós Graduação em Saúde da Criança e do Adolescente em nível de Mestrado e Doutorado da Faculdade de Medicina da Universidade Federal do Rio Grande do Sul.Pesquisador 1C Cnpq. Chefe do Centro de Pesquisa Clinica do Hospital de Clinicas de Porto Alegre, com grande experiência em cuidado neonatal e em pesquisa com seres humanos. Com publicações em pesquisa clinica em Neonatologia explorando o mecanismo fisiopatológico das doenças e os diversos desfechos que envolvem o seguimento de prematuros, com foco no crescimento de desenvolvimento.

Colaborador (co-investigador)

T. Michael O'Shea, MD, MPH: Professor de Pediatria e epidemiologista da Universidade da Carolina do Norte, Chapel Hill .Chefe da Unidade de Neonatologia e Medicina Perinatal, Carolina do Norte Chapell Hill, Estados Unidos. Coordenador do ELGAN-2 estudo vinculado com a Rede Americana de Pesquisa com apoio do NICHD. Mais de 300 publicações no pubmed. Professor O'Shea auxiliará na orientação e na supervisão das medidas de avaliação, dos desfechos, auxiliando ainda na análise crítica das possibilidades de emprego desse programa de intervenção precoces realizado pelos pais em outros locais com condições sócio econômicas desfavoráveis.

VI Citações

Procianoy RS, Mendes EW, Silveira RC. Massage therapy improves neurodevelopment outcome at two years corrected age for very low birth weight infants. Early Hum Dev. 2010; 86 (1):7-11.

Silveira RC, Procianoy RS. High plasma cytokine levels, white matter injury and neurodevelopment of high risk preterm infants: assessment at two years. Early Hum Dev. 2011 Jun;87(6):433-7.

Silveira RC, Fortes Filho JB, Procianoy RS. Assessment of the contribution of cytokine plasma levels to detect retinopathy of prematurity in very low birth weight infants. Invest Ophthalmol Vis Sci. 2011 Mar 10;52(3):1297-301.

Oliveira MG, Silveira RC, Procianoy RS .Growth of very low birth weight infants at 12 months corrected age in southern Brazil. J Trop Pediatr. 2008 ;54(1):36-42.

Filipouski GR, Silveira RC, Procianoy RS. Gestational Age on Neurodevelopment of Very Low-Birth-Weight Preterm Infants Am J Perinatol 2013;30:673–680.

Procianoy RS, KochMS, Silveira RC. Neurodevelopmental outcome of appropriate and small for gestational age very low birth weight infants. J Child Neurol 2009;24: 788–794.

T. Michael O'Shea, L. Corbin Downey, Karl K. C. Kuban Extreme prematurity and attention deficit: epidemiology and prevention Front Hum Neurosci 2013; 7: 578.

Seetha Shankaran, Aiping Lin, Jill Maller-Kesselman, Heping Zhang, T. Michael O'Shea, Henrietta S. Bada, Jeffrey R. Kaiser, Richard P. Lifton, Charles R. Bauer, Laura R. MentMaternal race, demography and health care disparities impact risk for IVH in preterm neonates J Pediatr. Author manuscript; available in PMC 2015 May 1.

Silveira RC, Filipouski GR, Goldstein DJ, O'Shea TM, Procianoy RS Agreement between Bayley Scales second and third edition assessments of very low-birth-weight infants. Arch Pediatr Adolesc Med. 2012; 166(11):1075-6.

2. Visão geral de tomada de decisão planos e processos

Uma vez atingido os quatro objetivos da pesquisa, ao final de dois anos; s próximos passos, caso houvesse financiamento disponível, seria continuar avaliando o neurodesenvolvimento dessa coorte até a idade pré-escolar, com finalidade de determinar o impacto dessa intervenção precoce, na janela de oportunidade da maior plasticidade da criança prematura, em longo prazo. A maior inovação proposta consiste em estabelecer o momento ótimo de intervenção continuada envolvendo cuidadores, medir esse efeito e posteriormente avalia a manutenção do mesmo, reduzindo as morbidades psiquiátricas mais prevalentes em prematuros, possivelmente devido ao controle do ambiente domiciliar, capacitando para a aprendizagem em nível escolar e para adultos futuros com melhor preparo e inserção social.

VII Apêndices

A. Planilha de Orçamento

Recursos humanos	Ano 1	Ano 2
Psicológa(o)s	R$32480,00	R$ 32.200,00
Fonoaudiólogas	R$411,00	R$ 15.600,00
Fisioterapeutas	R$14.000,00	R$23.800,00
Nutricionista-avaliação	-	R$21000,00
Passagens para apresentação de trabalhos em Congressos Internacionais	R$39000,00	R$39000,00
Enfermagem/terapeuta ocupacional	R$ 25.000,00	R$ 21.000,00
Transporte para visitas domiciliares	R$32340,00	R$ 8.400,00
Maleta de testes AIMS e Bayley III	R$14589,00	-
Computadores (2)	R$ 6.000,00	-
Material para estimulação precoce	R$ 13540,00	-
Treinamento da equipe	R$ 1200,00	-
Encontros de Capacitação	R$ 38000,00	-
Material gráfico com impressos	R$4788,00	-
Bolsistas de Iniciação Cientifica(apoio técnico)	R$39600,00	R$39600,00
Custos administrativos FundaçãoMédica do RS (10%)		R$25000,00
Total estimado/ano	R$ 260.948,00	R$ 225600,00
Total do projeto	=	R$486548,00

B. Detalhamento Narrativo do Orçamento

O custo do projeto envolve basicamente recursos humanos de ordem multiprofissional para realização da intervenção em ambiente hospitalar e ambulatorial antes da alta da UTI Neonatal (treinamento, reuniões de capacitação para a pesquisa) e garantia de acompanhamento da equipe multidisciplinar nos momentos das consultas de seguimento (braço 1 da randomização) e esse procedimento acrescido da instituição de um programa de orientação sistematizada aos familiares para realização de estímulos no domicilio (braço 2). Além dos custos dos profissionais com as medidas de desfechos. Os investigadores principais (IP) estarão presentes nessas etapas da pesquisa, tanto na internação neonatal (RSP) quanto no seguimento ambulatorial (RCS).

Capacitação da equipes para as intervenções: serão vinte encontros em módulos de orientações para toda a equipe multiprofissional. Aluguel de sala (35 lugares) com equipamento áudio visual no valor de R$ 1900,00/encontro. Total estimado R$38000,00

Visitas domiciliares O custo das visitas domiciliares envolvem transporte de toda a equipe que fará a monitorizaçao dos cuidadores em intervalos entre as consultas, totalizando 10 visitas/paciente.

É estimado um total de 420 visitas domiciliares (de acordo com cálculo amostral) com transporte da equipe mutidisciplinar, em área de abrangência de 60 km do centro de atendimento a R$ 32,00 ida e R$ 45,00 retorno.

Total Transporte para visitas domiciliares R$32.340,00 no primeiro ano

Total Transporte para visitas domiciliares R$8400,00, no segundo ano

Reuniões prévias com treinamento da equipe na randomização na neonatologia, para coleta de dados e aplicação do questionário de avaliação dos vínculos (BDI); realizado pelos neonatologistas (3) . Os pesquisadores farão a orientação dos bolsistas e toda equipe de pesquisa.

Bolsista de Apoio Técnico em extensão no país (ATP) R$ 550,00/mês por bolsista (6 bolsistas AT /ano): R$= 39600,00 no primeiro ano e R$= 39600,00 no segundo ano.

Neonatologistas: Coordenação das equipes de pesquisa na neonatologia e no seguimento ambulatorial, Os investigadores principais apresentarão resultados parciais e finais em Congressos Internacionais: R$39500,00/ano.
Fisioterapeutas: farão sessões de orientação às mães em todos os momentos das intervenções no seguimento (10 sessões/sujeito do GI, totalizando 420 orientações sistemáticas no seguimento, sendo 280 no primeiro ano e 140 no segundo ano).Total R$14000,00/ano 1 e R$ 7000,00/ano 2 .
O custo total para aplicações da escala AIMSpor fisioterapeutas treinadas estimado é de um total R$ 16.800,00; para ambos os grupos (GC e GI), sendo que deverão ser cegadas para qual grupo o sujeito pertence. Custo total da assistência fisioterápica será R$ 37800,00.
Enfermagem/terapeuta ocupacional: avaliação do cuidado materno, incentivo ao aleitamento materno, cuidado canguru são parte do manejo assistencial. Enfermeiras da pesquisa que irão orientar/supervisionar mães diariamente a partir de 48 horas de vida, randomizadas para GI e com reuniões clinicas receberão R$18000,00 no primeiro ano. Terapia ocupacional que irá orientar alguns exercícios passivos irá receber R$ 7000,00 no primeiro ano. Esses valores incluem todos os sujeitos alocados no grupo de intervenção até o momento da alta hospitalar. Custo total dessa atividade: R$25000,00. No seguimento, participarão do grupo multiprofissional com orientações para melhora do cuidado e supervisão da estimulação precoce realizada pelas famílias nas 10 intervenções e nas consultas de seguimento. A terapeuta ocupacional fará uma série de exercícios motores nas visitas domiciliares. O custo estimado para essa atividade é enfermagem (2) R$ 18000,00 e da terapeuta ocupacional (1) R$ 3000,00.
Psicóloga (o): em número de três, sendo um para realizar as testagens e avaliações do neurodesenvolvimento e dois para realizar as orientações de intervenções psicoafetivas, cognitvas e comportamentais aos pais; preparo das mães para a alta hospitalar, supervisão e avaliações dos vínculos parentais, capacitações junto à equipe multidisciplinar. O valor de cada pacote de orientação na Neonatologia será R$ 4200,00/42 sujeitos do grupo intervenção para cada profissional. Avaliações dos vínculos (PBI) serão R$ 120,00/sujeito de ambos os grupos. Totalizando R$ 10080,00. Custo total na internação=R$18480,00. Farão orientação às mães em todos os momentos das intervenções no seguimento (10 sessões/sujeito do GI, totalizando 420 orientações sistemáticas no seguimento, sendo 280 no primeiro ano e 140 no segundo ano).Total R$14000,00/ano 1 e R$ 7000,00/ano 2 .
O custo total para aplicações das escalas Bayley, congnição, linguagem e motora, por psicóloga capacitada e treinada é um total estimado de R$ 25200,00.
Fonoaudióloga (o): acompanhamento na rotina assistencial na neonatologia, com duas orientações (uma a cada semestre ao grupo de pesquisa) no custo total de R$ 411,00 . No seguimento ambulatorial farão parte da equipe multiprofissional que orientará para estimulação e para visitas domiciliares no grupo intervenção, realizando estimulações precoce no eixo da linguagem. O custo estimado para essa atividade é R$ 15600,00
Os custos dos materiais para estimulação da criança e aplicação dos testes, profissional que os aplica e o próprio kit dos testes será detalhado abaixo:
Materiais para estimulação:

Móbile colorido um para cada sujeito incluído no Grupo Intervenção;total= R$ 1260,00
Bichos de borracha;
R$ 4,00 a unidade, total: R$ 504,00
Chocalho colorido sem luz e com luz R$ 15,00 (sem luz) e R$ 50,00 (com luz e som), total= R$ 2730,00
Bola grande plástica
Total R$ 420,00
Luvas de lã preto e branca
Total= R$ 210,00
Bolas pequenas coloridas de borracha atóxica
R$ 3,50 (unidade). Total=:R$ 588,00
Espelho pequeno , um para cada sujeito incluído no Grupo Intervenção
Total; R$ 210,00
Livros com desenhos de bichos e objetos dois para cada sujeito do grupo intervenção
Total=R$ 1512,00
Livros com estórias, dois para cada sujeito do grupo intervenção
Total=R$ 1260,00
Material EVA 1 m x 1m, um para cada sujeito incluído no Grupo Intervenção
Total=R$ 420,00
Boneca de pano,

Total=R$ 1080,00
Brinquedos de encaixar, coloridos (cubos, formas geoméricas). R$ 30,00 (unidade).
Total =R$ 2260,00
Bastões de madeira com 60 cm de tamanho cada ,
R$ 25,00 (conjunto).Total= R$ 1050,00
Total geral do material para estimulação: R$ 13504,00

Material gráfico com impressos
Material gráfico e de computação para digitação dos dados da internação e do seguimento e para orientação aos pais (folders informativos), (10 por visita estipulada, sendo 2 coloridos/vez)
Folder R$ 1,00 (preto e branco) e R$ 3,00 (colorido). Serão entregues 8 preto e branco e dois coloridos para cada sujeito incluído no GI, com custo de R$ 14,00/paciente. Total R$ =588,00.
Kit de impressos das curvas de crescimento (84), três curvas em cada grupo; convencional e intervenção
Cada Kit custa R$ 6,00/ Unidade (252 kits) Total R$=1512,00
Kit de impressos da Escala AIMS (84), uma aplicação em cada grupo; convencional e intervenção
Cada kit custa R$ 12,00 /Unidade. Total R$=1008,00
Impressos do questionário PBI (84), duas aplicações em cada grupo; convencional e intervenção, incluindo folhas de tutorial e escores.
Cada Kit custa R$ 10,00 Unidade. Total R$1680,00
Total do material gráfico com impressos R$ 4788,00

Montagem do banco de dados, dupla digitação, análises estatísticas
R$= 13200,00 (bolsistas de iniciação cientifica) no segundo ano
Avaliações nutricionais
Na neonatologia, o manejo nutricional será realizado pelo próprio *staff* do serviço de nutrição, não gerando custos. Os bolsistas irão registrar velocidade de crescimento nas curvas de referencia, usando os dois computadores com um programa estatístico apropriado para avaliação nutricional (ANTHRO).
No seguimento, haverá uma nutricionista do grupo multidisciplinar que fará as visitas domiciliares com a equipe e mais duas nutricionistas para realizar as avaliações de crescimento nos momentos exatos em cada grupo (cegada para qual grupo a criança foi previamente alocada).
Total avaliação nutricional, R$21.000,00

DVD da escala AIMS (a ordem de solicitação da compra deve ser mediante contato institucional em carta timbrada ou formulário próprio da instituição). DVD U$ 140,00 e custo do envio U$ 10,00; total U$ 150,00
(R$= 468,00*). O bloco de 50 folhas com baterias de testes custa U$ 95,90/UNIDADE (R$ 300,00*).Nós necessitaremos pelo menos quatro baterias (total de R$ 1200,00)
Escalas bayley (BSDI III): Kit Abrangente com maleta de rodinha e escore assistencial. Inclui Manual de Administração, Manual Técnico, 25 formulários de registro / Idioma / avaliações da motricidade, linguagem e cognição de forma independente. Livro de estímulos, livro ilustrado, material completo para brincar e
interagir durante a avaliação de 6 meses a 36 meses de idade corrigida; 25 questionários acerca dos aspectos sócio-emocionais/ Comportamentais Adaptativo, 25 relatório sobre o que o cuidador efetivamente faz em estimulações. DVD com fundamentos da Administração da escala, *checklist* observacional . Assistente Administrativo (gravaçoes eletrônicas de pontuações, simulando exemplos de casos), e um DVD de Fundamentos da administração . Custo U $ 1592,00 (R $ 4968,00 *).
Bayley ™ -III Registro do formulário com kit de Combinação Cognitiva / Idioma / Motor , o pacote com 25 = U$ 169,0 / unidade (4 pacotes= U $ 676,00) Custo total R$ 2.110,00 *
Bayley ™ -III escala Motora: Registro do formulário com kit de dados da motricidade ampla e fina; pacote com 25 = U$ 107,00 / unidade (4 pacotes= U$ 428,00). Custo total de R$1336,00 *
Bayley ™ -III: Registro do formulário com kit de questionários sócio-emocional e Comportamental Adaptativo, o pacote com 25 = U$144,00 / unidade (4 pacotes= U$ 576,00) . Custo total de R$ 1798,00
* Bayley ™ -III: Formulário com Relatório do cuidador principal (mãe, pai, avós conforme o caso). pacote com
25 = U$107,00 / unidade (4 pacotes= U$ 428,00) . Custo total de R$ 1335,00 *
Bayley ™ -III: custo do livro de orientação para estímulos apropriados de 0 a 36 meses de idade corrigida = U$ 406,00 / Unidade .R$1.267,00 *
Bayley ™ -III: custo do livro de gravuras = U $ 34,00 / Unit.R$ 107,00 *

O custo total dos instrumentos para aplicação das escala Bayley é R$12.921,00 * (* Valor calculado de câmbio em 2015/05/25)
Total de equipamentos/instrumentos para AIMS e Bayley III escalas (um ano): R$14589,00

Qualificações do examinador para aplicar a escala AIMS: Deve ser administrado por profissionais de saúde da área infantil que tenham conhecimento de desenvolvimento motor normal infantil e experiência com a administração do instrumento. O nosso custo total para aplicações por fisioterapeutas treinadas estimado é de um total R$ 16.800,00; para ambos os grupos (GC e GI).
Computadores (2); sendo um para uso exclusivo de anotações e observações realizadas durante a internação neonatal e posteriormente nas visitas domiciliares. Total :R$ 6.000,00

Sumário do orçamento:

Bolsas: R$ 79.200,00 (6 bolsas ATP - A por 24 meses)

Capital: R$ 20.589,00

Custeio: R$ 386.759,00

Total: R$ 486.548,00

C <u>Referencias Bibliográficas</u>

1. March of Dimes, PMNCH, Save the Children, WHO. Born too Soon: The global action report on preterm birth. World Health Organization. Geneva, 2012.

2. Guinsburg R, de Almeida MF, de Castro JS, Silveira RC, Caldas JP, Fiori HH, ET AL. Death or survival with major morbidity in VLBW infants born at Brazilian neonatal research network centers.J Matern Fetal Neonatal Med. 2015; 2:1-5.
3. Spittle A, Orion J, Anderson P, Boyd R, Doyle LW. Early developmental intervention programmes post-hospital discharge to prevent motor and cognitive impairments in preterm infants. Cochrane Database of Systematic Reviews 2012; issue 12 Art n :CD005495.
4. Melnyk BM, Alpert-Gillis L, Feinstein NF, Fairbanks E, Czarniak-Schultz N, Hust D, et al. Improving cognitive development of low-birth-weight premature infants with the COPE program: a pilot study of the benefit of early NICU intervention with mothers. Research in Nursing and Health 2001; 24(5):373–89.
5. Field T. Preterm infant massage therapy studies: an American approach. Semin Neonatol 2002; 7: 487–494.
6. Bond C. Positive touch and massage in the neonatal unit: a British approach. Semin Neonatol 2002; 7: 477–486.
7. Litmanovitz I, Dolfin T, Friedland O, Arnon S, Regev R, Shainkin-Kestenbaum R et al. Early physical activity intervention prevents decrease of bone strength in very low birth weight infants. Pediatrics 2003; 112: 15–19.

8. Mendes EW, Procianoy RS. Massage therapy reduces hospital stay and occurrence of late-onset sepsis in very preterm neonates .J Perinatol 2008; 28:815-830.

9. Terra L, Hauck S, Schestatsky S, Fillipon AP, Sanchez P, Hirakata V, Ceitlin LH. Confirmatory factor analysis of the Parental Bonding Instrument in a Brazilian female population. Aust N Z J Psychiatry. 2009 ;43(4):348-54.
10. Procianoy RS, Mendes EW, Silveira RC. Massage therapy improves neurodevelopment outcome at two years corrected age for very low birth weight infants. Early Hum Dev. 2010; 86 (1):7-11.
11. Valentini NC , Saccani R. Escala Motora Infantil de Alberta: validação para uma população gaúcha . Rev Paul Pediatr 2011; 29 (2):231-8.
12. Bayley N. Manual for the Bayley Scales of Infant and Toddler Development. 3rd ed. San Antonio, TX: Psychological Corporation; 2006
13. Spittle AJ, Lee KJ, Spencer-Smith M, Lorefice LE, Anderson PJ, Doyle LW. Accuracy of Two Motor Assessments during the First Year of Life in Preterm Infants for Predicting Motor Outcome at Preschool Age. PLoS One. 2015;10(5):e0125854.
14. Marlow N. Neurocognitive outcome after very preterm birth. Arch Dis Child Fetal Neonatal Ed 2004;89:F224–F228.
15. Follow-up care of high-risk infants. Pediatrics 2004; 114:1377– 1397.
16. Vohr BR, Wright LL, Poole WK, McDonald SA. Neurodevelopmental outcomes of extremely low birth weight infants.
17. Anderson P, Doyle LW; Victorian Infant Collaborative Study Group. Neurobehavioral outcomes of school-age children born extremely low birth weight or very preterm in the 1990s. JAMA 2003;289: 3264–3272.
18. Morgan C, Novak I, Badawi N. Enriched environments and motor outcomes in cerebral palsy: systematic review and meta-analysis.Pediatrics 2013;132 (3):e735-46.
19. Morgan C, Novak I, Dale RC, Badawi N. Optimising motor learning in infants at high risk of cerebral palsy: a pilot study.BMC Pediatr. 2015 Apr 1;15:30. doi: 10.1186/s12887-015-0347-2.
20. Morgan C, Novak I, Dale RC, Guzzetta A, Badawi N.GAME (Goals - Activity - Motor Enrichment): protocol of a single blind randomised controlled trial of motor training, parent education and environmental enrichment for infants at high risk of cerebral palsy.BMC Neurol. 2014 Oct 7;14:203. doi: 10.1186/s12883-014-0203-2.
21. Roberts MY, Kaiser AP, Wolfe CE, Bryant JD, Spidalieri AM.Effects of the teach-model-coach-review instructional approach on caregiver use of language support strategies and children's expressive language skills.J Speech Lang Hear Res. 2014 ; 57(5):1851-69.
