# Supplementary material for: Parent-Guided Developmental Intervention for Infants With Very Low Birth Weight: A Randomized Clinical Trial
Source: JAMA Netw Open. 2024 Jul 17;7(7):e2421896. doi: 10.1001/jamanetworkopen.2024.21896 (PMC11255911; doi:10.1001/jamanetworkopen.2024.21896)
Supplement: Supplement 2. — eTable 1. Neonatal Outcomes for Randomized Participants eTable 2. Maternal Practice and Knowledge and Home Opportunities eTable 3. Comparison of Cognitive, Language, and Motor Scores for Enhanced Developmental Intervention and Usual Care Groups eTable 4. Research Activities for Enhanced Developmental Intervention and Usual Care Groups eFigure. Longitudinal Changes: BSDI-III Cognitive, Language, and Motor Composite Scores for EDI and UC Groups [file jamanetwopen-e2421896-s002.pdf]

Supplementary Online Content

Silveira RC, Velentini NC, O'Shea TM, et al. Parent-guided developmental intervention for infants with very low birthweight: a randomized clinical trial. *JAMA Netw Open*. 2024;7(X):e2421896. doi:10.1001/jamanetworkopen.2024.21896

eTable 1. Neonatal Outcomes for Randomized Participants

eTable 2. Maternal Practice and Knowledge and Home Opportunities

eTable 3. Comparison of Cognitive, Language, and Motor Scores for Enhanced Developmental Intervention and Usual Care Groups

eTable 4. Research Activities for Enhanced Developmental Intervention and Usual Care Groups

eFigure. Longitudinal Changes: BSDI-III Cognitive, Language, and Motor Composite Scores for EDI and UC Groups

This supplementary material has been provided by the authors to give readers additional information about their work.

© 2024 Silveira RC et al. *JAMA Network Open*.

eTable 1. Neonatal Outcomes for Randomized Participants

outcome	Enhanced Developmental Intervention Group (n=50)	Usual Care Group (n=50)
Blood transfusions	1.7 (2.4)	2.2 (2.6)
Days hospitalized in NICU	79.2 (40.4)	65.7 (30.8)
Days of invasive mechanical ventilation	6.8 (13.7)	7.6 (15.6)
Days of non-invasive mechanical ventilation	2.8 (4.0)	4.6 (6.5)
Days on continuous positive airway pressure	6.3 (7.2)	5.9 (6.6)
Days on supplemental oxygen	13.5 (21.5)	8.3 (11.3)
Days on parenteral nutrition	19.4 (23.7)	20.1 (18.6)
Days to full enteral nutrition	24.7 (24.8)	22.3 (19.3)
Days to regain birth weight	9.2 (3.7)	11.3 (7.9)
Length of Hospital Stay (days)	48 (14)	53 (15)
Late-onset sepsis	13 (26)	19 (38)
Periventricular hemorrhage	17 (34)	18 (36)
Periventricular leukomalacia	2 (4)	2 (4)
Previous preterm birth	4 (8)	8 (16)
Pulmonary hemorrhage	5 (10)	2 (4)
Bronchopulmonary Dysplasia	8 (16)	6 (12)
Neonatal resuscitation†	43 (90)	45 (90)
Surfactant use	25 (50)	35 (70)
NICU seizures	8 (16)	11 (22)
Daily weight gain (grams)	23.2 (6.0)	22.1 (6.6)
Weigh at hospital discharge (grams)	2563 (485)	2712 (627)
Length at hospital discharge (centimeters)	45.1 (2.7)	46.2 (3.0)
Head circumference at hospital discharge (centimeters)	33.4 (1.5)	34.0 (1.6)

Data are means (standard deviation in parenthesis) or number of participants (percentage of group in parenthesis) except were noted. None of the group differences are statistically significant at α level of 0.05.

Abbreviations: NICU – neonatal intensive care unit

† neonatal resuscitation refers to support with either CPAP or T-piece resuscitator in delivery room

© 2024 Silveira RC et al. *JAMA Network Open*.

eTable 2. Maternal Practice and Knowledge and Home Opportunities

DAIS, & KIDI & AHEM at 12 months		Groups M(SD)		Mean difference (95% CI)
		**EDI**	**UC**	
**DAIS**	**Daily Active Infant Scale**			
	Feeding	2.5 (0.7)	2.3 (0.8)	0.2 (-0.2 to 0.58)
	Bath	2.5 (0.7)	2.4 (0.8)	0.1 (-0.28 to 0.48)
	Changing	2.3 (0.8)	2.2 (0.8)	0.1 (-0.35 to 0.55)
	Carried by caregiver	2.5 (0.7)	2.4 (0.7)	0.1 (-0.36 to 0.56)
	Calm play	2.4 (0.8)	2.4 (0.8)	0.0 (-0.41 to 0.41)
	Active play	2.3 (0.8)	2.4 (0.8)	-0.1 (-0.49 to 0.29)
	Outside activity	2.3 (0.8)	2.3 (0.7)	0.0 (-0.50 to 0.50)
	Sleep	2.2 (0.9)	2.3 (0.7)	-0.1 (-0.61 to 0.41)
	**Total Score**	18.9 (6.0)	18.4 (5.7)	0.5 (-2.32 to 3.32)
**KIDI**	**Parents Knowledge**	0.63 (0.1)	0.62 (0.1)	0.01 (-0.05 to 0.07)
**AHEM**	**Home Affordances for Development**	19.7 (11.0)	19.1 (12.8)	0.6 (-5.57 to 6.77)

Note. M; Mean; SD: Standard Deviation; CI: Confidence Interval.

© 2024 Silveira RC et al. *JAMA Network Open*.

eTable 3. Comparison of Cognitive, Language, and Motor Scores for Enhanced Developmental Intervention and Usual Care Groups

Neurodevelopment Outcomes: BSITD-III Composite Scores & AIMS percentiles		Groups M(SD) EDI	Usual Care	Mean difference (95% CI)
**BSID-III Composite**				
**Cognitive**	4 months	100.7 (14.7)	93.5 (14.8)	**7.2 (0.4 to 14.0)**
	8 months	100.4 (9.7)	92.6 (14.7)	**7.8 (2.2 to 13.4)**
	12 months	103.7 (12.8)	97.6 (14.1)	6.1 (-0,4 to 12.6)
	18 months	101.8 (11.9)	97.3 (13.5)	**4.5 (0.1 to 8.9)**
**Language**	4 months	96.1 (14.0)	89.9 (13.3)	**6.2 (0.5 to 11.9)**
	8 months	101.2 (15.7)	90.9 (12.7)	**10.3 (5.1 to 15.6)**
	12 months	100.4 (14.1)	92.3 (15.8)	**8.1 (2.1 to 14.0)**
	18 months	99.3 (12.0)	91.6 (14.4)	**8.3 (2.6 to 14.1)**
**Motor**	4 months	98.8 (15.4)	92.8 (16.4)	6.0 (-1.2 to 13.3)
	8 months	95.9 (12.3)	86.7 (15.9)	**9.2 (3.7 to 14.7)**
	12 months	98.4 (13.7)	87.9 (18.1)	**10.5 (4.0 to 17.1)**
	18 months	102.5 (13.3)	92.7 (17.9)	**9.8 (3.2 to 16.5)**
**AIMS Motor Percentiles**				
	4 months	31.0 (21.3)	25.7 (24.1)	5.3 (-5.4 to 15.9)
	8 months	41.3 (26.6)	26.0 (23.5)	**15.3 (6.2 to 24.3)**
	12 months	52.2 (23.5)	29.4 (22.9)	**22.8 (15.6 to 30.1)**
	18 months	53.7 (17.0)	30.3 (21.0)	**23.4 (16.8 to 30.0)**

Abbreviations: M: Mean; SD: Standard Deviation; CI: Confidence Interval

© 2024 Silveira RC et al. *JAMA Network Open*.

eTable 4. Research Activities for Enhanced Developmental Intervention and Usual Care Groups

**Time**	**Intervention activity**	**Enhanced Developmental Intervention**	**Usual Care**
7th day in NICU until discharge	tactile-kinesthetic stimulation by mothers	x	
	infant massage	x	x
NICU discharge through 6 months corrected age	monthly follow-up visits	X	X
	Home visits by developmental specialists: instructions for parents, demonstration of physical therapy interventions and age-appropriate developmentally supportive acitvities	x	
6 months corrected age through 12 months	Bimonthly clinic visits	X	X
	Home visits by developmental specialists: instructions for parents, demonstration of physical therapy interventions and age-appropriate developmentally supportive acitvities	x	
After 12 months corrected age	Every 3 months clinic visits	x	X
18 months corrected age	Primary outcome assessed	x	x

© 2024 Silveira RC et al. *JAMA Network Open*.

eFigure. Longitudinal Changes: BSDI-III Cognitive, Language, and Motor Composite Scores for EDI and UC Groups

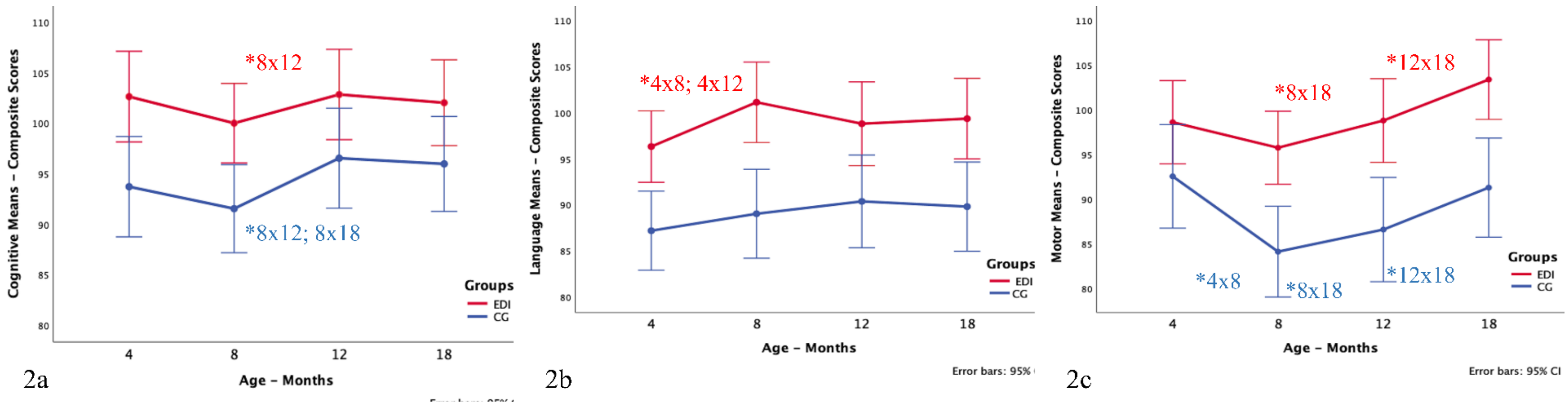

EDI: Enhanced Developmental Intervention; UC: Usual Care; BSID-III: Bayley Scales of Infant and Toddler Development version III.

© 2024 Silveira RC et al. *JAMA Network Open*.
