# Supplementary material for: Parent-Guided Developmental Intervention for Infants With Very Low Birth Weight: A Randomized Clinical Trial
Source: JAMA Netw Open. 2024 Jul 17;7(7):e2421896. doi: 10.1001/jamanetworkopen.2024.21896 (PMC11255911; doi:10.1001/jamanetworkopen.2024.21896)
Supplement: Supplement 3. — Data Sharing Statement [file jamanetwopen-e2421896-s003.pdf]

Data Sharing Statement

Silveira. Parent-Guided Developmental Intervention for Infants With Very Low Birth Weight. *JAMA Netw Open*. Published July 17, 2024. doi:10.1001/jamanetworkopen.2024.21896

Data
Data available: Yes
Data types: Other (please specify)
Additional Information: Unidentified study data may be shared upon request for the reserchers.
How to access data: drarita.c.s@gmail.com
When available: With publication

Supporting Documents
Document types: Statistical/analytic code, Informed consent form
How to access documents: drarita.c.s@gmail.com
When available: With publication

Additional Information
Who can access the data: anyone requesting the data
Types of analyses: for a specified purpose
Mechanisms of data availability: after approval of a proposal
